# A robust ultra-microporous cationic aluminum-based metal-organic framework with a flexible tetra-carboxylate linker

**DOI:** 10.1038/s42004-023-00938-x

**Published:** 2023-07-06

**Authors:** Shyamapada Nandi, Asma Mansouri, Iurii Dovgaliuk, Philippe Boullay, Gilles Patriarche, Ieuan Cornu, Pierre Florian, Georges Mouchaham, Christian Serre

**Affiliations:** 1 Institut des Matériaux Poreux de Paris, Ecole Normale Supérieure, ESPCI Paris, CNRS, PSL University, 75005 Paris, France; 2 Chemistry Division, School of Advanced Sciences, Vellore Institute of Technology, 600127 Chennai, India; 3 Normandie Université, ENSICAEN, UNICAEN, CNRS, CRISMAT, 14050 Caen, France; 4 Université Paris-Saclay, CNRS, Centre de Nanosciences et de Nanotechnologies, 91120 Palaiseau, France; 5 Centre National de la Recherche Scientifique (CNRS), UPR3079 CEMHTI, Université d’Orléans, 1D Av. Recherche Scientifique, CEDEX 2, 45071 Orléans, France

**Keywords:** Metal-organic frameworks, Characterization and analytical techniques, Porous materials

## Abstract

Al-based cationic metal-organic frameworks (MOFs) are uncommon. Here, we report a cationic Al-MOF, MIP-213(Al) ([Al_18_(*μ*_2_-OH)_24_(OH_2_)_12_(mdip)_6_]6Cl·6H_2_O) constructed from flexible tetra-carboxylate ligand (5,5'-Methylenediisophthalic acid; H_4_mdip). Its crystal structure was determined by the combination of three-dimensional electron diffraction (3DED) and high-resolution powder X-ray diffraction. The structure is built from infinite corner-sharing chains of AlO_4_(OH)_2_ and AlO_2_(OH)_3_(H_2_O) octahedra forming an 18-membered rings honeycomb lattice, similar to that of MIL-96(Al), a scarce Al-polycarboxylate defective MOF. Despite sharing these structural similarities, MIP-213(Al), unlike MIL-96(Al), lacks the isolated *μ*_*3*_-oxo-bridged Al-clusters. This leads to an ordered defective cationic framework whose charge is balanced by Cl^-^ sandwiched between two Al-trimers at the corner of the honeycomb, showing strong interaction with terminal H_2_O coordinated to the Al-trimers. The overall structure is endowed by a narrow quasi-1D channel of dimension ~4.7 Å. The Cl^-^ in the framework restrains the accessibility of the channels, while the MOF selectively adsorbs CO_2_ over N_2_ and possesses high hydrolytic stability.

## Abstract (web summary)

Aluminium-based cationic metal–organic frameworks remain rare, yet offer opportunities for unusual framework structures and material properties. Here, a robust ultra-microporous cationic metal–organic framework based on aluminium building units and flexible tetracarboxylic acid linkers is reported, and three-dimensional electron diffraction combined with high-resolution powder X-ray diffraction show that the material comprises an ordered defective cationic framework with narrow quasi-1D channels.

## Introduction

Metal-Organic frameworks (MOFs) constructed from inorganic nodes (metal ions, clusters, chains, layers, etc.) linked via organic linkers have emerged as a new class of porous solids. Owing to their ordered porous structure, highly tunable pore size, shape, and composition, they have been proposed for a wide range of potential applications such as separation^1–8^, gas storage^9,10^, catalysis^11–13^, heat reallocation^14,15^, water harvesting^16^, biomedicine^17,18^ etc. Among different classes of MOFs reported so far, trivalent (Al, Cr, Fe) and tetravalent (Ti, Zr, Hf, Ce) metal polycarboxylate MOFs are considered as the most promising candidates due to their high hydrolytic stability compared to different divalent metal carboxylate MOFs^19–23^.

**Fig. 1:**
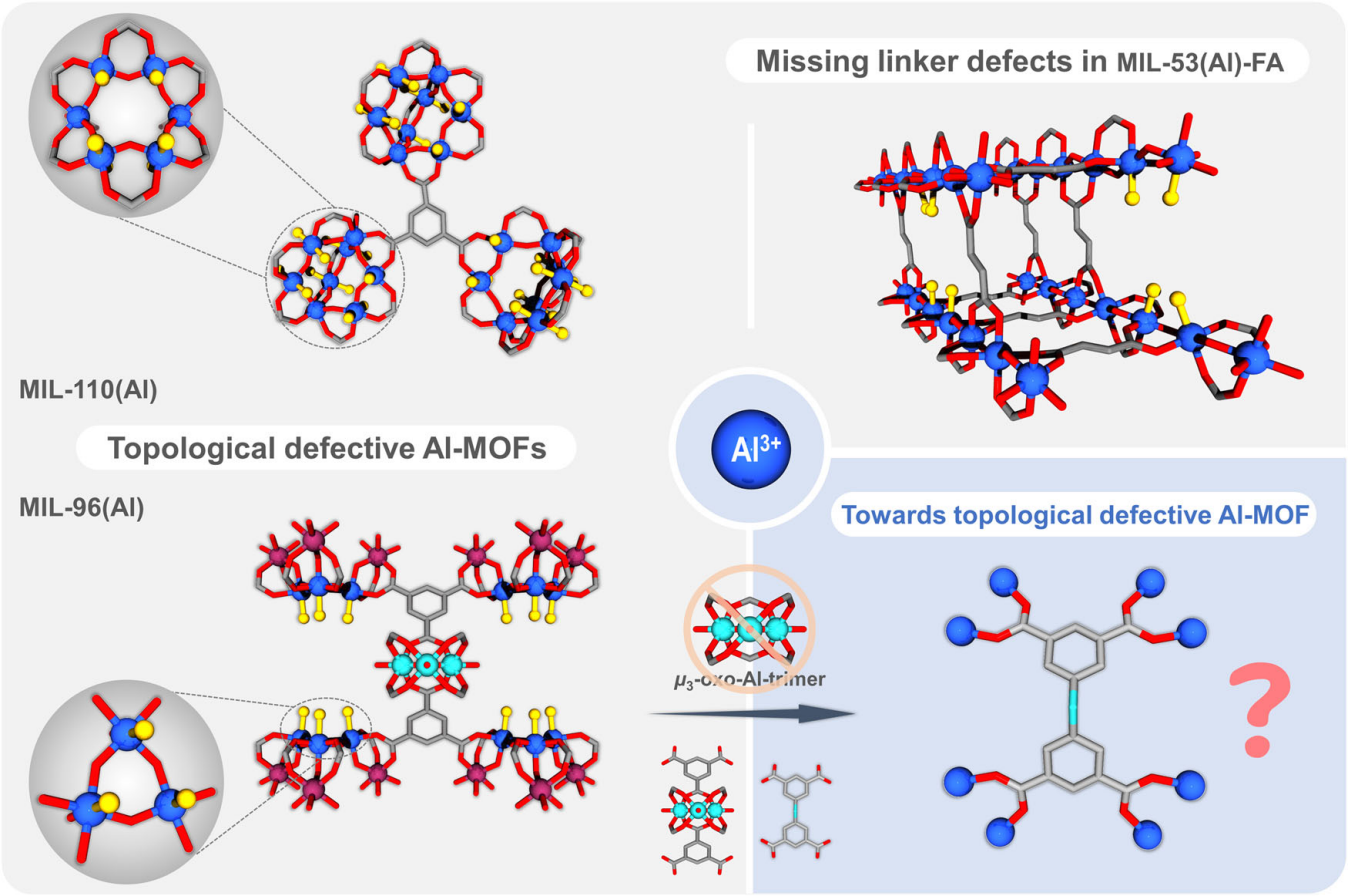
Al-defective MOFs. Examples of Al-MOFs showing topological defects and missing linkers as well as our new proposed synthesis strategy to generate a topologically defective MOF inspired by MIL-96(Al) structure.

Defects in MOFs, that break the systematic arrangements of the atoms or motifs within the crystal of the parent framework, has become an important additional tool for tuning their properties. These are mainly related to the organic moieties but can also concern the inorganic building units. In the case of ligand defects, one can distinguish (i) topological defects where usually no bridging ligands are expected as per the topological requirement; and (ii) missing linkers where normally a topologically needed ligand is absent in the structure. In both cases, the absence of bridging ligand can be compensated by the coordination of solvents, modulators, or other labile ligands. Thus, both the defects create some potential coordination vacancies around the adjacent metal/clusters and thereby drastically change the properties of the solids. Very recently, defective MOFs have attracted tremendous attention due to their unique properties compared to the non-defective MOFs^24–26^. Via engineering the defects, and hence the potentially uncoordinated sites, one can provide novel opportunities in adsorption^26,27^, catalysis^28^, optical^29^, magnetic^29^, and conducting properties^27^, etc. Indeed, defect engineered M^IV^-MOFs enabled enhanced properties compared to the respective non-defective analogs, such as in catalysis^30–32^, pollutant or organic dye removal^33,34^, heat allocation^35^, and adsorption^26,27,36,37^. More importantly, ligand defect has a significant impact on Lewis and Bronsted acidities^38^ of the solids giving rise to unique binding/active and/or molecular imprinted sites^28^. The proton conductivity of the MOFs could also be tuned eventually boosted via grafting of acidic species to the defect sites^39,40^. It is quite common to observe defects in case of a tetravalent metal-based MOFs such as Zr/Hf/Ce-based MOFs. Although these defective MOFs, particularly the Zr-based ones, show very promising properties, controlling the defects onto the SBUs is not a trivial task especially when synthesized in large scale in the presence of a modulator. Similar to Zr/Hf-based MOFs, very few Ti-based defective MOFs could also be observed^41,42^. When it turns to the case of trivalent metals-based MOFs such as those based on Fe^III^, several defective Fe-MOFs has been observed particularly when the MOFs are derived from the Fe-based trimeric oxo/hydroxo clusters^43,44^. Interestingly, in the case of Al-MOFs, there exist only very few examples of defective MOFs^45^. The reason for this is that the chemistry of Al-MOFs can be quite complex to control leading often to different polymorphs. Besides, in most of the cases, the structures are built from Al-hydroxo chains where it is more challenging to introduce defects (Fig. 1). In contrast, the latter are, in general, more frequently observed in the case of oxo/hydroxo-cluster-based MOFs, although very scarce in the case of Al-MOFs. For instance, Al-trimesates such as MIL-96(Al) and MIL-110(Al) exhibit topological defects leading to the presence of terminal hydroxyl groups and water molecules^46,47^. Indeed, MIL-96(Al) comprises oxo-centered Al-trimers that are coordinated to bridging trimesates, as well as another 18-member 2D hexagonal secondary building unit made from infinite chains of AlO_4_(OH)_2_ and AlO_2_(OH)_4_ octahedra (arranged in a trimeric fashion). The structure contains terminals water molecules as well as terminal hydroxyl groups which potentially act as open metal sites (OMS) or coordinatively unsaturated sites, that might be also used as grafting sites for active metal ions or nanoparticles. These hydroxyls groups could also be replaced by other anions (such as chloride, fluoride, nitrate, or acetate depending on the reactants used in the synthesis) as well. Despite such a possibility, it has not been exploited except in the case of fluoride^48^. Due to its highly functional active sites (terminal water and OH groups), this MOF has been explored for several applications such as CO_2_ capture^49,50^, catalytic dye degradation^51^, liquid phase absorption of *p*-hydroxybenzoic acid^51^, fluoride removal from water^48^, etc. Series of Al-MOFs derived from di/tri/tetra-carboxylate linkers have been reported to date. However, reports on Al-polycarboxylate MOFs be either cationic and/or defective remain still extremely rare^46,47^. For example, the typical dicarboxylates such as terephthalate (1,4-BDC)^52^, isophthalate (1,3-BDC)^53^, fumarate (FA)^54^ and their derivatives led to rigid or flexible frameworks without any topological defect. Only missing linker defects have been reported such as in the case of MIL-53-FA associated with the presence of Lewis acid sites^54^. In case of tricarboxylates, although few trimesate MOFs exhibited structural defects, the use of benzene-1, 3, 5-tribenzoate (BTB)^55^ does not lead to any structural defect. Similarly, different tetra carboxylates such as 1,2,4,5 benzene tetracarboxylate (BTeC)^56,57^, 1,2,4,5-tetrakis-(4-carboxylatophenyl)-benzene (TCPB)^58^, tetrakis(4-carboxyphenyl) porphyrin (TCPP)^59^, 1,3,6,8-tetrakis (*p*-benzoic acid) pyrene (TBAPy)^59^, and biphenyl tetra carboxylate (BPTC)^60^, led only to rigid or flexible framework without any topological defect. In most cases, one can conclude that when the use of rigid linkers is considered, one ends up with rigid/flexible MOFs without any topological defects, albeit scarce exceptions. Therefore, the idea here was to explore the use of flexible linker for the construction of topologically defective Al-MOFs inspired by MIL-96(Al) structure.

In this regard, herein we investigated the flexible tetracarboxylic acid linker (5, 5'-Methylenediisophthalic acid; H_4_mdip; Supplementary Fig. 1) for the construction of new Al-MOFs. After screening a wide range of synthesis conditions (solvent, pH, temperature, metal sources, etc.), we finally obtained a new ultra-microporous MOF having a general formula [Al_18_(*μ*_2_-OH)_24_(OH_2_)_12_(mdip)_6_]6Cl·6H_2_O denominated as MIP-213(Al), cationic in nature (MIP stands for Materials from Institute of Porous Materials of Paris). Its crystalline structure was determined using a combination of three-dimensional electron diffraction (3DED) and high-resolution Powder X-ray diffraction (HRPXRD). The MOF exhibits a hexagonal structure very similar to MIL-96(Al) framework as a consequence of similar inorganic secondary building unit (SBU) built from the infinite chains of AlO_4_(OH)_2_ and AlO_2_(OH)_3_(H_2_O) octahedra forming a honeycomb lattice based on 18-membered rings, without, however, the isolated oxo-centered Al-trimers in the case of MIP-213(Al). Consequently, MIP-213(Al) possesses chloride ions which are sandwiched between two topologically defective Al-trimers present at the corner of the honeycomb. Due to the presence of these anions, the pores along [1 0 0] become inaccessible for nitrogen at 77 K. To assess the potential of this MOF for adsorption of small gas molecules, and as this compound possesses very high hydrolytic stability of interest for real applications, we have explored the selective adsorption of CO_2_ over N_2_ at room temperature, taking into account the promises of MIL-96(Al) for the CO_2_ capture^45^.

## Results and discussion

MIP-213(Al) was synthesized under ambient pressure conditions employing green solvents. In a typical synthesis, H_4_mdip linker was dissolved in equivalent amount of 2 M NaOH and added to AlCl_3_ ∙ 6H_2_O dispersed in a mixture of benzyl alcohol which was preheated to 180 °C. The mixture was then refluxed at 200 °C overnight. After cooling the mixture to room temperature, the solid off-white powder was isolated via filtration followed by washing with water, ethanol and drying the sample at 80 °C. The dried solid product was characterized using PXRD, thermogravimetric analysis (TGA), FTIR, and other advanced characterization methods (Supplementary Figs. 2–7).

### Structural characterization and analysis

Because of the small size of the obtained crystallites (ca.100–300 nm, Supplementary Figs. 8-9), we have first attempted to solve the structure from HRPXRD methods. The indexing of the HRPXRD pattern revealed that the material crystalizes in a hexagonal crystal system with space group *P*6_3_/*mmc (N° 194)*. However, our initial crystal structure determination from the HRPXRD data was not successful. In addition, our attempts to grow single crystals yielded above the submicronic scale, even under solvothermal conditions, were not successful (Supplementary Figs. 8 and 9), ruling out the structure determination from single crystal X-ray diffraction even under a microfocus synchrotron source. We then considered the assistance of single crystal electron diffraction technique known as 3DED^61,62^ which allows obtaining structure solution from tiny crystals. The continuous rotation electron diffraction (cRED)^63^ data has been collected using the instrumental conditions detailed in supporting information (“Material and methods” section). The temperature of the sample in the TEM was decreased to 95 K using a cryo-transfer holder, to enhance the stability of the MOF under the electron beam. Two datasets (collected in less than 20 s) were selected to be merged into a single dataset with an improved combination of resolution and completeness.

The structure solution step gives a 3D density map with well-identified positions for all non-hydrogen atoms (Supplementary Fig. 11). Actually, only a strong residual density present in the voids of the structure seemed to us more difficult to attribute with certainty and was initially thought to be free water molecules. Nonetheless, the presence of water molecules did not match with the observation made from the TGA data (Supplementary Fig. 6) and, therefore, we had to consider other possibilities for the inclusion of solvent molecules to the voids of this framework. STEM imaging of the MOF’s particles combined with EDX spectroscopy provides a map of the elemental distribution and indicates the presence of chloride ions homogeneously in the sample (Supplementary Fig. 11). The presence of this halide arises from the salt AlCl_3_ used as a precursor for the MOF synthesis. From the initial density map obtained from 3DED data after the structure solution step (Supplementary Fig. 11a), replacing water molecules with Cl^−^ ions is in good agreement with the highest residual intensity (Supplementary Fig. 12). While the 3DED data were of good quality to provide a structural model, the kinematical refinement convergences to high *R* factors (*R*(obs) = 33.25%, wR(obs) = 33.15) which prevented from any finer structural analysis. The obtained model was thus confirmed using a Rietveld refinement against the laboratory HRPXRD data (Supplementary Fig. 12 and Supplementary Data 1), where satisfying *R* factors were obtained (*R*_Bragg_ = 13.1%, *R*_F_ = 9.01%). The inclusion of the Cl^-^ to the voids of the framework resulted in a better match between the experimental and calculated diffraction patterns compared to inclusion of water molecules_._

**Fig. 2:**
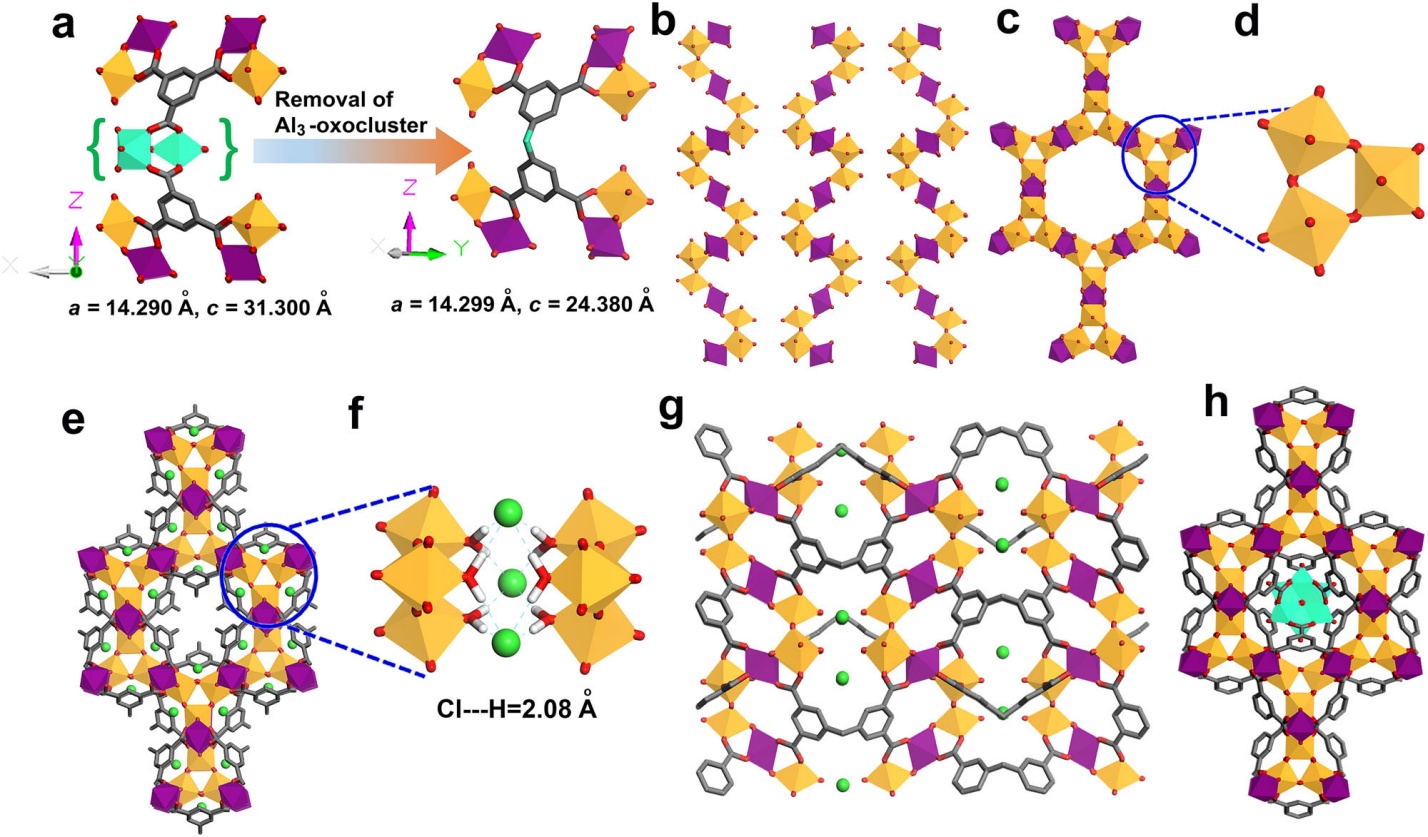
Depiction of the crystal structure of MIP-213(Al) and the structural similarities with MIL-96(Al). **a** The geometrical similarities of the framework structures of MIL-96(Al) and MIP-213(Al). **b** Secondary building unit, the infinite chains of AlO_4_(OH)_2_ and AlO_2_(OH)_3_(H_2_O) octahedra present in MIP-213(Al). **c** Inorganic SBU along [0 0 1] showing the infinite chains of AlO_4_(OH)_2_ and AlO_2_(OH)_3_(H_2_O) octahedra forming an 18-membered hexagonal honeycomb layer. **d** Al-trimers present in the corner of the hexagonal honeycomb layers. **e** 3D view of the crystal structure of MIP-213(Al) along [0 0 1], showing the narrow corrugated hexagonal channel. **f** Zoom-in view at the corners of the hexagonal honeycomb showing the exact position of the chloride ions with typical Cl…H interaction. **g** 3D view of the crystal structure along [1 0 0]. **h** 3D view of the crystal structure of MIL-96(Al) along [0 0 1]. Color code: AlO_4_(OH)_2_ polyhedra, AlO_2_(OH)_3_(H_2_O) polyhedra, carbon, oxygen, and chloride atoms are in purple, orange, gray, red, and light-green, respectively (*μ*_3_-oxo-centered Al-trimers presented by the cyan polyhedral; hydrogen atoms are omitted for clarity).

The crystal structure of MIP-213(Al) (*P*6_3_/*mmc* with *a* = 14.2995(3) Å) shows strong similarities with the structure of MIL-96(Al) (*P*6_3_/*mmc* with *a* = 14.290 Å). Only, the *c* parameters of both crystal structures differ (31.300(6) versus 24.3801(10) Å for MIL-96(Al) and MIP-213(Al), respectively) in agreement with the change of the framework connectivity along [0 0 1]. From the geometrical considerations, the exclusion of the *μ*_3_-oxo-centered isolated Al-trimers from the structure of MIL-96(Al) and the “merging” of its two isophtalate linkers along [0 0 1] into one 5,5'-methylenediisophthalate are, as expected by replacing trimesate by mdip^4-^ linker, at the origin of the framework model of MIP-213(Al) (Fig. 2a).

The crystal structure analysis reveals that this MOF is built from the infinite chains of AlO_4_(OH)_2_ and AlO_2_(OH)_3_(H_2_O) octahedra forming a honeycomb lattice based on 18-membered rings which is very similar to the SBU present in MIL-96(Al) (Fig. 2). These SBU layers get connected by the tetracarboxylate linkers (mdip^4-^) and thereby generate the three-dimensional framework (Fig. 2e). The 3D framework consists of narrow hexagonal channels of dimension ~4.7 Å along [0 0 1] (Fig. 2e), which are interconnected with another narrow channels present along [1 0 0].

**Fig. 3:**
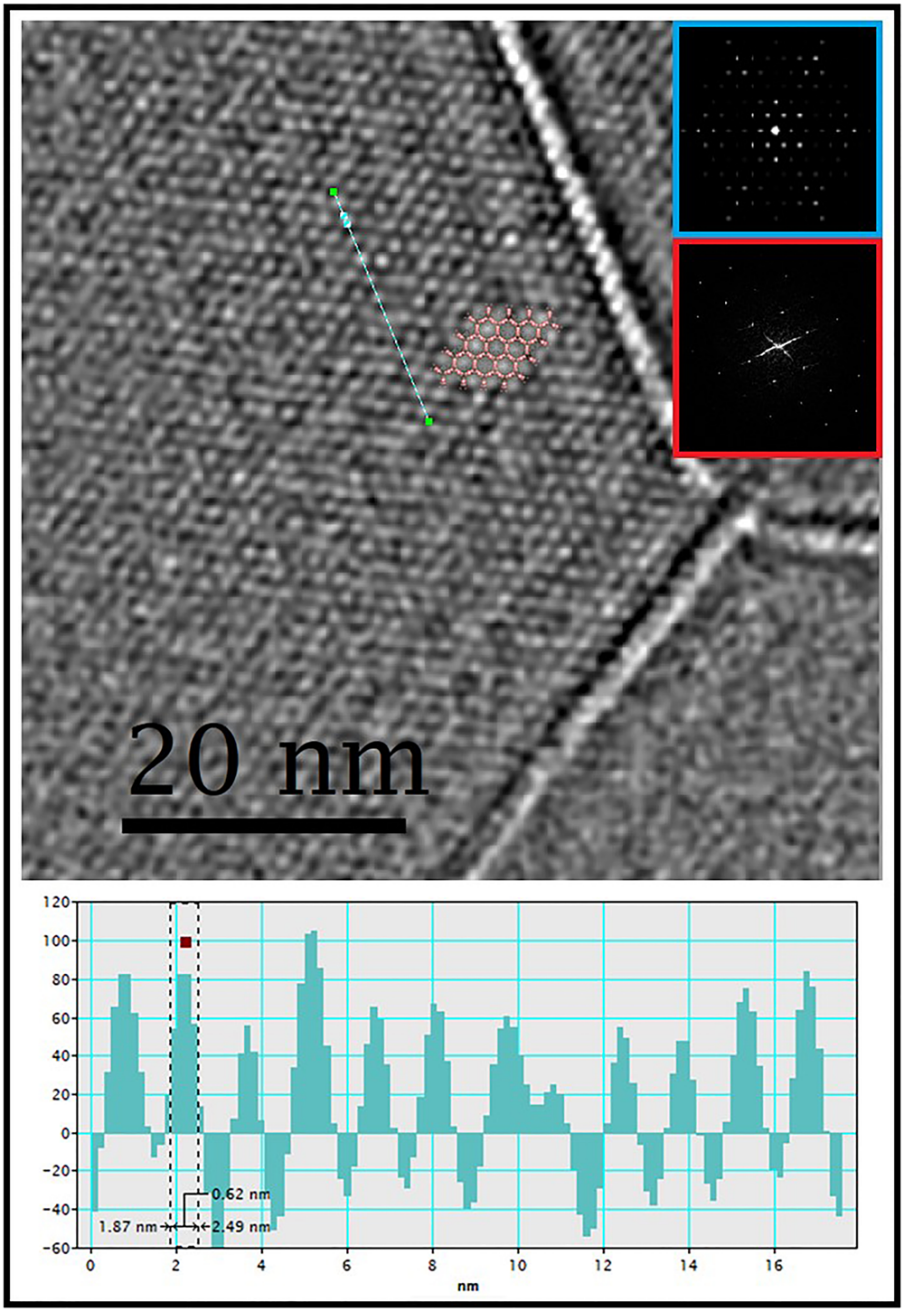
Low-dose HRTEM image of MIP-213(Al). Filtered HRTEM image of MIP-213(Al) oriented along the [0 0 1] axis. The structural model exhibiting the hexagonal channels along the *c* axis is superposed on the image. The insets are the FFT (red), the simulated electron diffraction pattern (blue), generated from the ED model using the Crystdiff software^74^.

According to the crystallographic structure, this framework shall be positively charged. The extra positive charge of the framework is here balanced by the presence of chloride counter ions which are typically positioned between two Al-trimers present at the corner of the honeycomb as presented in Figs. 2e, f. Interestingly, these chloride ions are having strong interaction with the terminal water molecules connected to the Al centers of the Al-trimers with a typical Cl….H distance of ~2.08 Å (Fig. 2f). Due to the presence of the chloride ions the channels/cages along [1 0 0] become very restricted for gas molecules to enter the channel (Fig. 2g), although such ultra-microporous windows might, as shown previously in the case of MFU-4 for instance, be accessible under specific conditions^64^. However, the hexagonal channels along the [0 0 1] are quite accessible to the gas molecules. Furthermore, the presence of channels of hexagonal symmetry was directly observed in real space using low-dose HRTEM imaging, from which the unit cell parameters (Supplementary Table 3) and the diameters of the channel are extracted, and the values corroborated well with those from the initial structural model from 3DED data (Fig. 3). The diameter of the channels calculated from the profile line is ~0.6 nm. The full width at half maximum (FWHM) of each peak is defined as the diameter of the corresponding channel (bright spot in the image; Fig. 3).

A comparative structural analysis shows very high topological similarities between the MIP-213(Al) and MIL-96(Al) (Fig. 2h and Supplementary Fig. 13). Both frameworks are built from the same 18-member hexagonal honeycomb layers from the infinite chains of AlO_4_(OH)_2_ and AlO_2_(OH)_3_(H_2_O)/AlO_2_(OH)_4_ octahedra (Supplementary Fig. 13). The main difference between MIL-96(Al) and MIP-213(Al) is the absence of the *μ*_3_-oxo-centered isolated Al-trimers in the later one. In the case of MIL-96(Al), if one removes the carboxylate groups connected to the isolated oxo-centered Al-trimers leading to an isophthalate, that accounts to one-half of the mdip^4-^ linker, then the generated SBU layer is the same as the SBU layer present in MIP-213(Al) (Supplementary Fig. 13a, d). Subsequently, in the case of the MIL-96(Al), these SBU layers are connected to each other via the third arm of the trimesate linker connecting the isolated Al-trimers (Supplementary Fig. 13g) whereas in the case of MIP-213(Al) the SBU layers are connected via the methylene carbon of the mdip^4-^ linker thereby generating the 3D framework (Supplementary Fig. 13h). The SBU layers are separated by a larger distance (O…O distance between the layers = 6.57 Å) compared to that observed in the case of MIP-213(Al) (O…O distance between the layers = 3.20 Å) (Supplementary Fig. 13). Another important difference is that MIL-96(Al) is neutral and contains terminal water/hydroxy ligands which are connected to the Al centers of the Al-trimers present at the corner of the hexagonal honeycomb layer. In MIP-213(Al) the framework exhibits a positive charge that is balanced by the chloride ions present in between the Al-trimers from two different layers (Supplementary Figs. 13j and 1e, f) while all the oxygen atoms connected to the Al-trimers at the corner of the hexagonal honeycomb are from the water molecules. Thus MIP-213(Al) possesses a hexagonal corrugated narrow channel along [0 0 1]. Nevertheless, in case of MIL-96(Al) this hexagonal channel is blocked by the isolated *μ*_3_-oxo centered Al-trimers (Supplementary Fig. 13i). Despite being cationic in nature, this framework is highly thermally stable under oxygen atmosphere as observed from the variable temperature PXRD and TGA measurement (Supplementary Figs. 5 and 6). This can be supported by the fact that the chloride counter ions do not easily leave the structure due to a strong interaction with the terminal water molecules. Overall, although the structures of MIP-213(Al) and MIL-96(Al) are obtained from 2 different ligands (pure organic for the former, and metalo-ligand for the latter), they are structurally quite well related thanks to the 18-membered hexagonal honeycomb layer SBU at the origin of both MOFs, with specific chemical features for each.

### Solid state NMR studies

Furthermore, for a deeper structural understanding solid-state NMR has been considered with ^1^H and ^13^C NMR to provide information on the linker’s structure whereas ^27^Al NMR is expected to shed light on the metal ions configurations. Supplementary figure 14 shows the ^1^H/^13^C two-dimensional correlation spectra evidencing the various proton and carbon environment and their connectivity. On the carbon side, intensities found around 130 ppm are assigned to the aromatic carbons (C4, C5, C14, C15) while some additional resonances found around 170 ppm and 40 ppm correspond to the carboxylates (C3) and methylene bridge (C17) carbons, respectively. To confirm this assignment, first principle calculations of NMR parameters were performed on the structure with proton positions optimized. The results are given in Supplementary Table 4 and show very reasonable agreement with the experimental spectrum (Supplementary Fig. 15). Additional resonances observed around 120 ppm and 60 ppm belongs to residual solvents (benzyl alcohol). Non-protonated carbons C3, C5, and C14 are further evidenced by their marked dependence to the contact time in the CPMAS experiments (Supplementary Fig. 16). This provides the full assignment of the six inequivalent carbons as shown in Supplementary Table 4. On the proton side, the 2D HETCOR along with the DFT calculations allows us to propose the assignment of the six inequivalent protons as shown in supplementary Fig. 17. The ^1^H signals absent from the ^1^H HETCOR projection correspond to the non-carbonated protons µ_2_-OH (H13 and H19) and structural H_2_O (H7), which are found, respectively, at around 2 ppm and 7 ppm, (Supplementary Fig. 18) in good agreement with the DFT calculation (Supplementary Table 4). An additional resonance is needed around 5 ppm and assigned to residual benzyl alcohol as it correlates in the ^13^C{^1^H} HETCOR to the carbon site assigned above to this residual solvent. Overall, the full assignment of the ^13^C and ^1^H spectra confirm the structural model of the linkers derived in the preceding sections. The ^27^Al spectra shown a complex lineshape resulting from overlapping sites partially resolved in an MQMAS experiments (Supplementary Figs. 19 and 20), showing three components with distribution of NMR parameters and, hence, distribution of structural environments. This 2D experiment can be satisfactory simulated with 1 site presenting a strong quadrupolar coupling constant (*C*_Q_ ~ 7.3 MHz) and 2 with rather small ones (*C*_Q_ ~ 2.5 MHz). The crystal structure predicts only two inequivalent aluminum sites (Al8 and Al9) and the DFT calculation shows that they possess contrasted *C*_Q_s: *C*_Q_(Al8) = 8.65 MHz and *C*_Q_(Al9) = 1.24 MHz, allowing to assign the site with *C*_Q_ ~ 7.3 MHz to Al8 of the form Al(OAl)_2_(OC)_4_. For the Al9 site of the form Al(OAl)_3_(OC)_2_(H_2_O), we expect a smaller *C*_Q_, but two sharp resonances are observed experimentally. A double-quantum 2Q-filtered experiment (Supplementary Fig. 21) which selects aluminum with close-by proximities, does remove the sharp line in the middle of the spectrum, this one being therefore attributed to an isolated aluminum arising from an impurity. The full 2Q/1Q experiment also evidence Al9/Al9 and Al9/Al8 proximities, and absence of Al8/Al8 proximities, in full agreement with the crystal structure. This allows attribution of the two resonances corresponding to the two inequivalent aluminum sites expected from the aforementioned structure and confirms the validity of the structure derived above.

### Gas sorption analysis

**Fig. 4:**
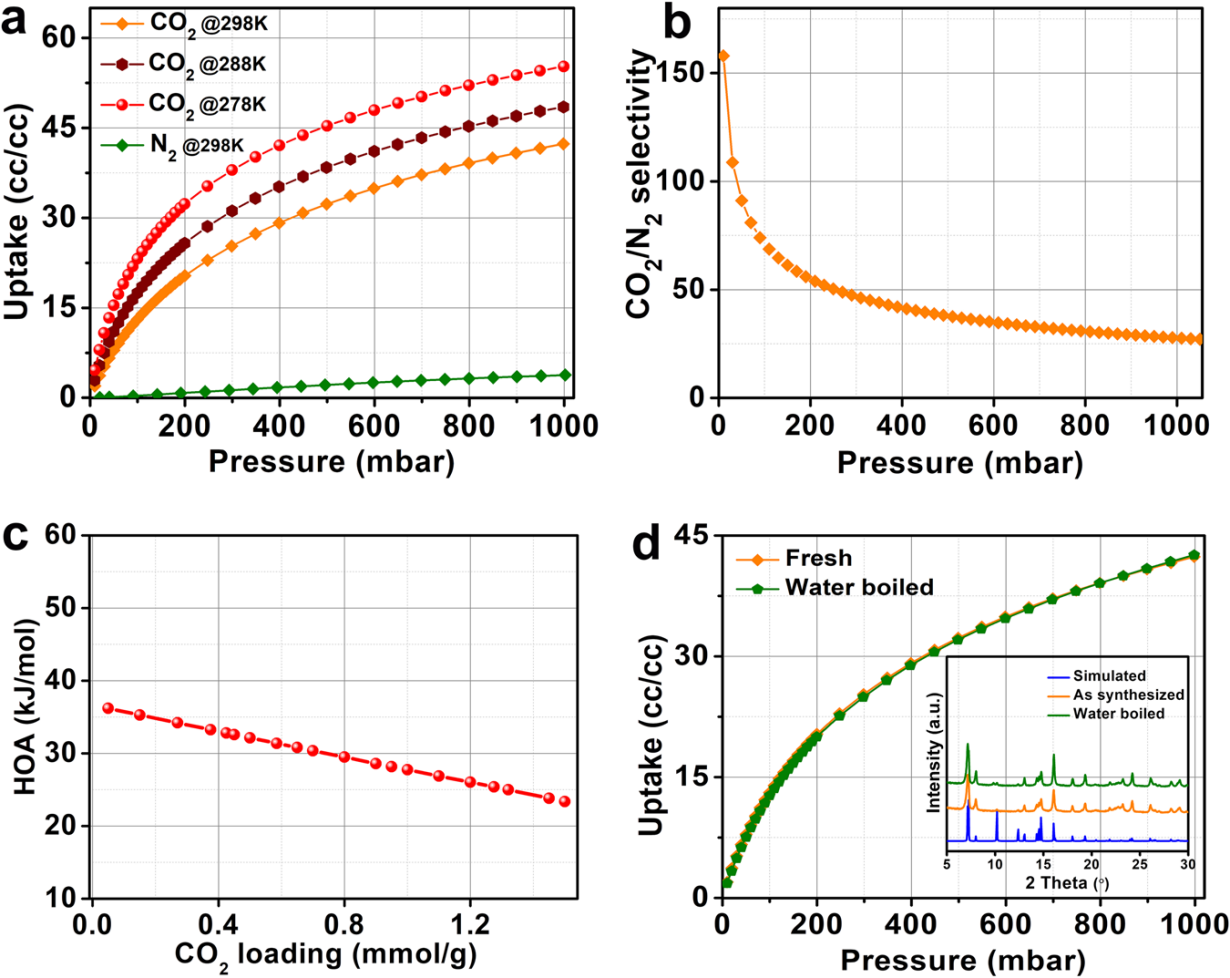
CO_2_ and N_2_ sorption data of MIP-213(Al) showing its selective CO_2_ capture property and high hydrolytic stability. **a** CO_2_ and N_2_ isotherms on MIP-213(Al) collected at different temperatures. **b** CO_2_/N_2_ selectivity predicted using IAST model employing a nominal composition of 15CO_2_:85N_2_. **c** HOA plot derived from a virial calculation employing CO_2_ isotherm at three different temperatures (278, 288, and 298 K). **d** A comparative 298 K CO_2_ isotherm of the fresh sample and water boiled sample showing the hydrolytic stability of the MOF. The inset shows the PXRD patterns (Cu K*α* radiation, *λ* = 1.5406 Å) of the fresh and water boiled sample.

As established from the structural investigation, the material possesses microporous channels. The permanent porosity of this MOF was established from the N_2_ sorption at 77 K (Supplementary Fig. 22). The material does not adsorb much N_2_ even at 77 K (saturation uptake ~1.0 mmolg^−1^) with a calculated BET surface area of 60 m^2^g^−1^, which is much lower compared to the one of MIL-96(Al) (>600 m^2^g^−1^). This can be explained in the case of MIP-213(Al), by the fact that the channels along [1 0 0] are inaccessible to N_2_ due to the presence of the chloride ions right at the center of the channels (details on ion exchange attempts are available in the Supplementary Information). Although, in case of MIP-213(Al) the channel along the [0 0 1] are open but the small channel diameter restricts the N_2_ molecule to diffuse. Considering the ultra-micropores present in MIP-213(Al), we further tested the CO_2_ capture properties of this MOF at room temperature (Fig. 4 and Supplementary Data 2). Interestingly at 298 K the material selectively adsorbs CO_2_ over N_2_ (CO_2_ uptake = 17.5 cc/cc at 0.15 bar against 3.8 cc/cc for N_2_ at 298 K and 1 Bar). Such a lower CO_2_ uptake of MIP-213(Al) compared to MIL-96(Al) was expected because of the presence of narrow channels partially occupied by the chloride ions in MIP-213(Al). An IAST prediction form the single component isotherms yielded a CO_2_/N_2_ selectivity of 60 (Fig. 4b and Supplementary Fig. 23) at 0.15 bar which is quite good in a view of post-combustion carbon capture applications. In fact, the selectivity value is slightly higher than some of the Al, Sc, and Zr-based microporous MOFs such as MIL-96(Al) (*S* = 40), ScBDC (*S* = 40), UiO-66(Zr) (*S* = 12), UiO-66(Zr)_BTeC (*S* = 30), UiO-66(Zr)_NH_2_ (*S* = 37) but comparable/slightly less than some of the microporous Al/Ti MOFs such as MIL-91(Al) (*S* = 68), MIL-91(Ti) (*S* = 150), MIL-69(Al) (*S* = 120)^49,65^.

Furthermore, a virial analysis of the CO_2_ isotherms at 273, 283, and 298 K yielded a zero coverage heat of adsorption (HOA) of 37 kJmol^−1^ (Fig. 4c and Supplementary Fig. 24). The water sorption isotherm at 298 K shows a clear type I isotherm indicating very strong interactions between the water molecules and the framework at low pressure (Supplementary Fig. 25). This is in good agreement with the structural model. One of the most important properties of MOFs to be implemented in any potential industrial applications is the robustness under humid atmosphere or the hydrolytic stability of the MOF^66^. Considering this we have subjected this MOF for stability tests under boiling water conditions. Interestingly, there was no sign of structural or chemical degradation even after 24 h of boiling as evidenced from the PXRD data (Fig. 4d) as well as CO_2_ adsorption isotherm on the water boiled sample, which further confirms the hydrolytic stability or robustness of this MOF (Fig. 4d).

In conclusion, based on a fine structural analysis and a “merging” strategy when replacing tricarboxylate by a flexible tetracarboxylate linker, we have developed a rare example of cationic Al-based MOF with topological defective sites. The structure elucidation from both 3DED and HRPXRD data for this MOF was complemented by low-dose HRTEM imaging and EDX chemical mapping. The MOF is built from the infinite chains of AlO_4_(OH)_2_ and AlO_2_(OH)_4_ octahedra which form 18-membered hexagonal honeycomb layers, similar to those present in MIL-96(Al). Although both MOFs share similar SBU, they strongly differ in some other chemical aspects. Unlike MIL-96(Al), MIP-213(Al) does not contain isolated *μ*_3_-oxo-centered Al-trimers leading to an overall cationic framework, where chloride ions balance the extra positive charge in the framework. Although the presence of these chloride ions in the framework makes this solid less porous (at least to N_2_ at 77 K) compared to MIL-96(Al), this MOF still selectively adsorb CO_2_ over N_2_ with a selectivity of 60 at 0.15 bar and 298 K. Moreover, the reported MOF possess high hydrolytic stability and could be applied for many other applications utilizing the defective/cationic nature and/or Lewis acidity. Considering the challenge of developing Al-MOFs with controlled defects, this study will pave the way for the design of new Al-based defective MOFs for versatile applications in near future.

## Methods

### Synthesis of MIP-213(Al)

#### Milligram-scale synthesis

MIP-213(Al) was synthesized in a typical ambient pressure reflux synthesis method employing AlCl_3_.6H_2_O and H_4_mdip linker, water, benzyl alcohol, and NaOH. In a typical synthesis, 0.723 g of AlCl_3_.6H_2_O (3 mmol) was dispersed in 18 ml of Benzyl alcohol and the mixture was heated to 180 °C. To this, a solution of H_4_mdip 0.345 g (1 mmol) in 2 ml of 2 M NaOH was added under continuous stirring. Additional 8 ml distilled water was added to the mixture. The reaction mixture was then refluxed at 200 °C for 16 h. After cooling the reaction to room temperature, the off-white powder was filtered and washed with 20 ml of water and 10 ml of ethanol. The final product was then air dried (yield = 440 mg). The powder product was then characterized using PXRD, IR, TGA, and adsorption analysis. The synthesis can be done in a different scale as per the requirement.

#### Gram-scale synthesis

In a typical synthesis, 7.23 g of AlCl_3_.6H_2_O (30 mmol) was dispersed in 180 ml of Benzyl alcohol and the mixture was heated to 180 °C. To this a solution of H_4_mdip 3.45 g (10 mmol) in 20 ml of 2 M NaOH was added under continuous stirring. Additional 80 ml distilled water was added to the mixture. The reaction mixture was then refluxed at 200 °C for 16 h. After cooling the reaction to room temperature, the off-white powder was filtered and washed with 100 ml of water and 60 ml of ethanol. The final product was then air dried (yield = 4.66 g).

Note: The synthesis temperature could be reduced up to 160 °C but with longer synthesis time (up to 48 h).

### Characterization of MIP-213(Al)

#### The crystal structure determination and refinement from HRPXRD

The unit cell parameters and the space group of MIP-213(Al) have been indexed using the program EXPO2014^67^. Although accurate cell parameters and the space group were determined from the indexing of the powder pattern, crystal structure determination was not successful (see supporting information for more details). The refinement using the Rietveld method was based on the model from 3DED using Fullprof^68^. The refinement of the powder X-ray diffraction data required the inclusion of benzyl alcohol solvent in the large cavities.

#### cRED data collection and structure determination

The cRED data were collected on a JEOL F200 cold-FEG operated at 200 kV, equipped with a GATAN RIO16 camera. A series of 2D electron diffraction patterns are acquired while continuously rotating the crystal using the Digital Micrograph plugin insteaDMatic^69^. The diameter of the parallel beam was set to 300 nm obtained by inserting a 10 μm condenser aperture. The sample was cooled down to a lower temperature (95 K) in the TEM using a Gatan Elsa™ cryo-transfer tomography holder, to reduce the electron beam damage.

Several cRED datasets were recorded and processed using PETS 2^70^. Two cRED datasets (Supplementary Table 1) collected on isolated crystals were kept to obtain the result presented here. These two datasets were merged in order to obtain a data completeness of 95.3% for a 0.71 Å resolution. The structure was solved using SUPERFLIP program (charge flipping method)^71^ and refined using JANA2020^72^ using electron scattering factors. The density maps (electrostatic potential map in Supplementary Fig. 11 and difference Fourier maps in Supplementary Fig. 12) are visualized using VESTA^73^. A summary of the data collection and structure refinement are given in Supplementary Table 2.

#### Low-dose high-resolution transmission electron microscopy

Low-dose HRTEM data were collected on the FEI Titan 80-300 E-TEM microscope equipped with the Gatan K2 direct-detection electron counting camera (DDEC), operated at 300 kV. An electron dose rate of 8 e^−^Å^−2^ is used. The “HRTEM filter” was used to apply a Wiener filter, followed by the Average Background Subtraction Filter (ABSF). The details to calculate the channel diameter is provided in the supporting information.

#### High-resolution scanning transmission microscopy

High-resolution scanning transmission microscopy images (HRSTEM) were acquired on a FEI Titan Themis microscope 200 corrected for spherical aberrations on the probe, operating at 200 kV, equipped with a Ceta 16 M hybrid camera from ThermoFischer Scientific capable of working under low-dose conditions.

#### Solid state NMR

^1^H, ^13^C and ^27^Al spectra were obtained on a Bruker Avance III 17.6T spectrometer operating at 750.0 MHz using magic angle spinning at 30 kHz. Direct spectra were obtained using a Hahn echo sequence with radiofrequency fields. Direct ^13^C NMR spectra were obtain using a CPMAS experiment.^27^Al was obtained using a quantitative pulse at ν_rf_ (^27^Al) = 50 kHz, and a recycle delay of 0.3 s based on estimated T1 measurements done by saturation-recovery experiments. 2Q/1Q ^27^Al-^27^Al correlation was used with a ${R2}_{1}^{2}$ recoupling (a detailed procedure is provided in the supporting information).

## Supplementary information

Supplementary Information

Description of Additional Supplementary Files

Supplementary Data 1

Supplementary Data 2

## Data Availability

Supporting information contains all the synthetic and experimental details as well as Supplementary methods (PXRD, N_2_, CO_2_ and water sorption, TGA, FTIR, etc.) and data. This material is available free of charge from https://www.nature.com/commschem/ or from the authors on request. The crystallographic structure reported in this study has been deposited at the Cambridge Crystallographic Data Centre (CCDC), under deposition number 2246502. CIF can be obtained free of charge from The Cambridge Crystallographic Data Centre or directly downloaded from the editor webpage as Supplementary Data 1. IAST and related numerical source data are available as Supplementary Data 2.
