# Supplementary material for: A robust ultra-microporous cationic aluminum-based metal-organic framework with a flexible tetra-carboxylate linker
Source: Commun Chem. 2023 Jul 6;6:144. doi: 10.1038/s42004-023-00938-x (PMC10326013; doi:10.1038/s42004-023-00938-x)
Supplement: Supplementary file 1 — Supplementary Information [file 42004_2023_938_MOESM1_ESM.pdf]

A Robust Ultra-microporous Cationic Aluminium-based Metal-Organic Framework with a Flexible Tetra-carboxylate Linker

Shyamapada Nandi,[1,2] Asma Mansouri,[1] Iurii Dovgaliuk,[1] Philippe Boullay,[3] Gilles Patriarche,[4] Ieuan Cornu,[5] Pierre Florian,[5] Georges Mouchaham,[1]* and Christian Serre[1]*

[1] Institut des Matériaux Poreux de Paris, Ecole Normale Supérieure, ESPCI Paris, CNRS, PSL University 75005 Paris, France.

[2] Chemistry Division, School of Advanced Sciences, Vellore Institute of Technology, 600127 Chennai, India.

[3] Normandie Université, ENSICAEN, UNICAEN, CNRS, CRISMAT, 14050 Caen, France.

[4] Université Paris-Saclay, CNRS, Centre de Nanosciences et de Nanotechnologies, 91120 Palaiseau, France.

[5] Centre National de la Recherche Scientifique (CNRS), UPR3079 CEMHTI, Université d'Orléans, 1D Av. Recherche Scientifique, CEDEX 2, 45071 Orléans, France.

Emails : georges.mouchaham@ens.psl.eu ; christian.serre@ens.psl.eu

1. Supplementary Materials, Methods and Instruments:

2. Supplementary Analytical Characterization:

PXRD analysis

TGA analysis

IR analysis

3. Supplementary Structure solution:

Electron diffraction, Microscopy study, XRD

4. Supplementary Solid State NMR:

5. Supplementary First-principle calculations:

6. Supplementary Gas/Water Sorption Analysis:

IAST Fitting

7. Supplementary Hydrolytic Stability:

8. Supplementary Note on Ion Exchange of The Material:

1. Supplementary Material and Methods:

All the chemicals were bought from Alfa Aesar and used without further purification.

Instrument Details and Methods.

PXRD: Routine Powder X-ray Diffraction (PXRD) data were collected using a high-throughput Bruker D8 Advance diffractometer working on transmission mode and fitted with a focusing Göbel mirror. The X-ray source was Cu-Kα radiation (λ = 1.5418 Å). High resolution Powder X-ray diffraction (HR-PXRD) data for structure solution was collected with sample sealed in 0.7 mm glass capillary with a PANalytical EMPYREAN diffractometer (CuKα1 radiation with Ge [1 1 1] monochromator, λ = 1.540598 Å) equipped with a PIXcel1D detector. Temperature dependent PXRD data were recorded with sample closely packed in quartz capillary on a PANalytical EMPYREAN diffractometer with CuKα radiation (λ = 1.5418 Å) and equipped with an HTK-1200N (Anton Parr) high-temperature chamber and a GaliPIX3D detector.

The crystal structure determination and refinement from (HRPXRD): The unit cell parameters and the space group of MIP-213(Al) have been indexed using the program EXPO2014.[1] The cell parameters are closely related to MIL-96(Al), which had similar unit cell parameters and the space group ($P6_3/mmc$).[2] Only, the *c* parameters of both crystal structures differ (31.300(6) versus 24.3791(7) Å for MIL-96(Al) and MIP-213(Al). Although we had accurate cell parameters from the indexing of the powder pattern, crystal structure determination was not successful. The refinement of the final crystal structure was performed using the model from 3DED in Fullprof software,[3] however it also required the inclusion of benzylalcohol solvent in the large cavities, which traces were suggested by the ssNMR. The trials to study of the desorbed sample were not successful due to low crystallinity of the compound after heating, see variable-temperature PXRD (Supplementary Fig. 5).

Low-dose high-resolution transmission electron microscopy:

The profile line drawn on a row of channels (observed as bright spots) enables us to calculate their diameter which is around 0.6 nm. The unit cell parameter *a* (equal to *b* in a hexagonal crystal system), is calculated from the distance between the family of planes 0,1,0. For d = 0.8 nm^{-1}, *a* = *b* = 14.309 ± 0.148 Å. The standard deviation was calculated from the full weight at half maximum (FWHM = 0.0197 nm^{-1}) of the Gaussian fit of the intensity profile's peak (Gaussian Fit script, D. R. G. Mitchell and W. Bertram, v 1.1, Mar 2004), using following equation.

$$SD = \frac{FWHM}{2\sqrt{(2ln2)}}$$

Energy dispersive X-ray spectroscopy:

The energy-dispersive X-ray spectroscopy (EDX) analysis allows performing chemical mapping using the "Super-X" systems for EDX analysis that equipped the microscope.

Sorption measurement:

Gas sorption experiments: Nitrogen sorption data at 77K was collected on a Micromeritics Tristar instrument. The CO_2, N_2 and H_2O isotherms at 298K were recorded on a Micromeritics Triflex instrument. In all the cases the measurements were recorded using ultra-high purity gases (≥4.8 grade). Prior to the adsorption measurement, the sample was degassed at (150°C) for 8 hours. The degassing was done in one step using a Micromeritics SmartVacPrep degas unit: evacuation at 150ºC on the degas port ($P=10^{-6}$ mbar), at which point the outgas rate was ≤ 2 μbar/min.

TGA: TGA data was collected on Mettler Toledo TGA/DSC 2, STAR System apparatus with a heating rate of 5 °C/min under the oxygen flow.

FTIR: Infrared spectra were measured with a Nicolet iS5 FTIR ThermoFisher spectrometer.

ssNMR: ^{1}H, ^{13}C and ^{27}Al spectra were obtained on a Bruker Avance III 17.6 T spectrometer operating at 750.0 MHz using magic angle spinning at 30 kHz. Direct spectra were obtained using a Hahn echo sequence with radiofrequency fields $\nu_{rf}(^{1}H)$ = 125 kHz and interpulse delay of 195 µs (6 rotor periods) and a recycle delay of 0.75 s based on estimated T1 measurements done by saturation-recovery experiments. Direct ^{13}C NMR spectra were obtain using a CPMAS experiment at $\nu_{rf}(^{13}C)$ = 20 kHz and $\nu_{rf}(^{1}H)$ = 125 kHz with a spinal64 decoupling at $\nu_{rf}(^{1}H)$ = 100 kHz for various cross-polarization contact time of 250 µs, 500 µs and 1000 µs and a recycle delay of 0.5 s.^{27}Al was obtained using a quantitative pulse at $\nu_{rf}(^{27}Al)$ = 50 kHz, and a recycle delay of 0.3 s based on estimated T1 measurements done by saturation-recovery experiments. 2Q/1Q ^{27}Al-^{27}Al correlation was use with a $R2_1^2$ recoupling at $\nu_{rf}(^{27}Al)$ = 10 kHz. (12 rotor periods) with ν_{rf} = 60 kHz, 27Al central-transition selective pulses at ν_{rf} = 10 kHz and 1H pulses at ν_{rf} = 100 kHz. External reference was TMS and a 1M solution of $Al(NO_3)_3$. All spectra were simulated using DMFit [4] using pseudo-Voigt function for ^{1}H and ^{13}C and second-order quadrupolar broadened line shapes for ^{27}Al under the assumption of infinite spinning speed.

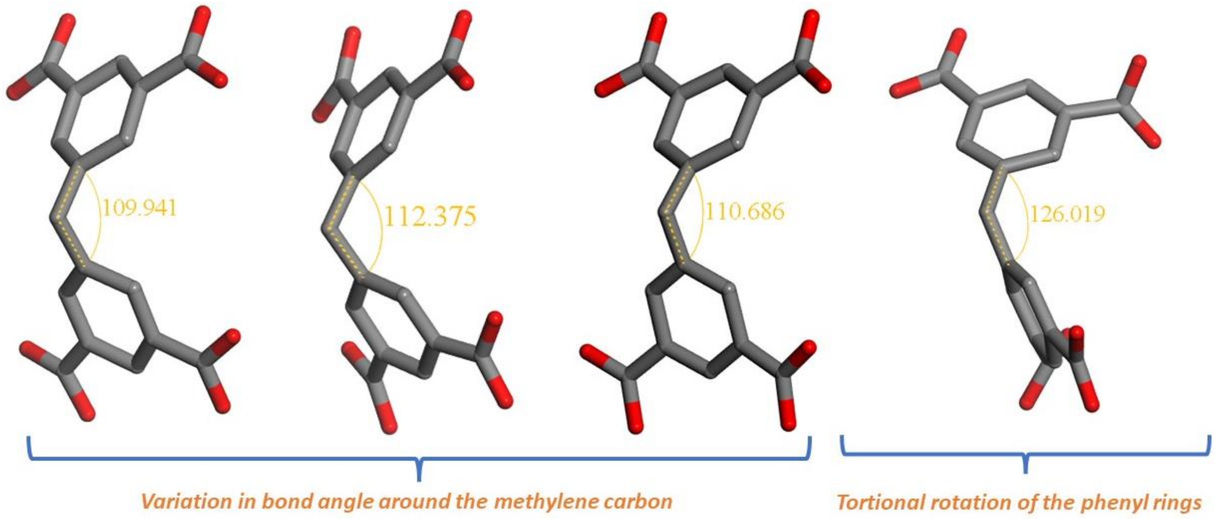

Supplementary Figure 1. Representations of the H_4mdip linker showing the flexibility associated with it.

2. Supplementary Analytical Characterization:

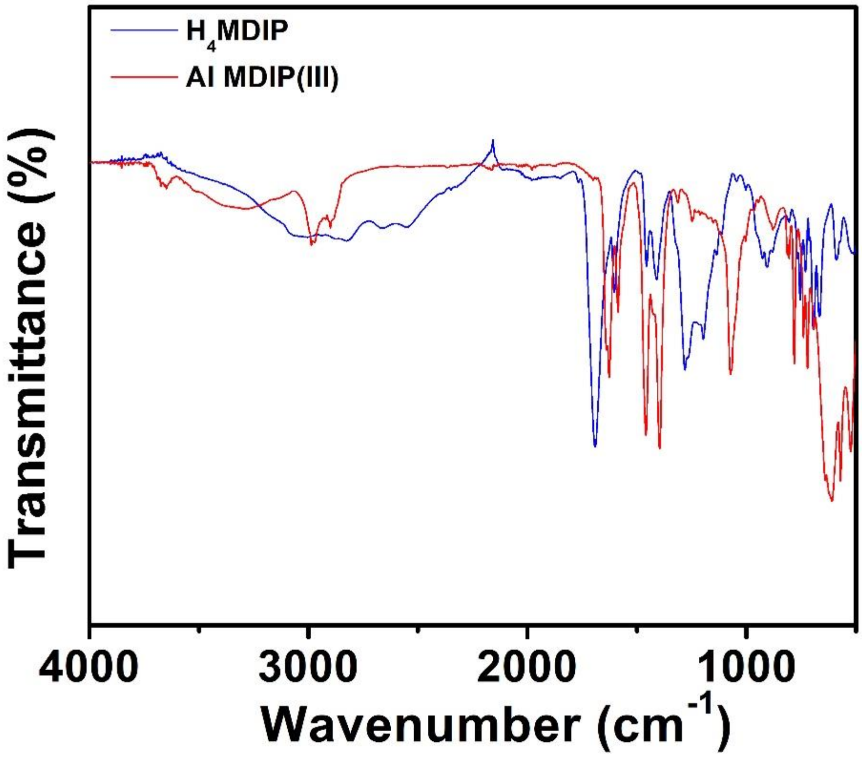

Supplementary Figure 2. FTIR spectra of MIP-213(Al) in comparison with the linker. This suggest that the sample is free from any linker inside the pore.

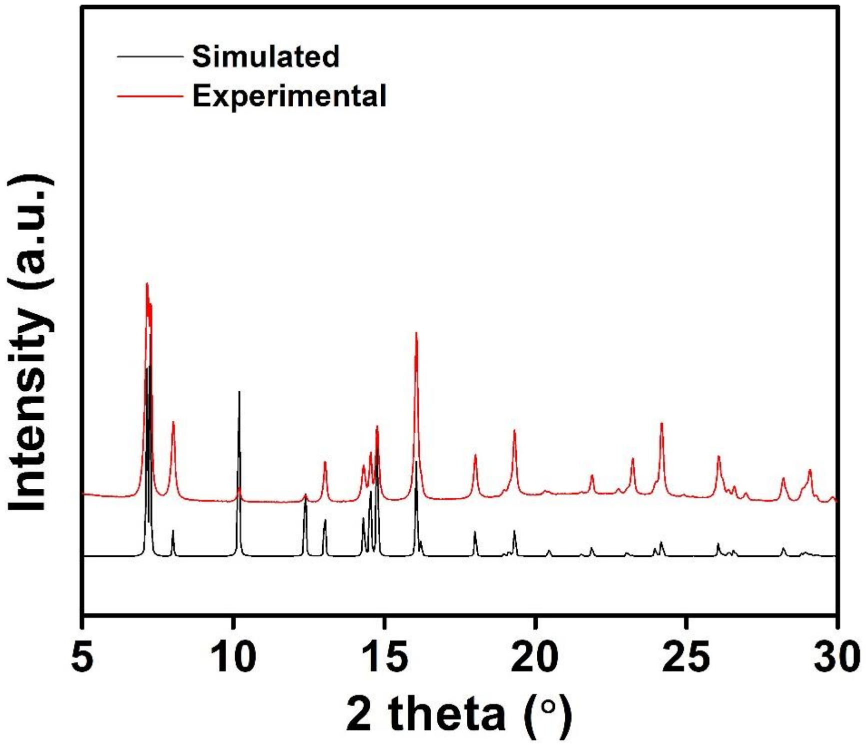

Supplementary Figure 3. A comparative PXRD plot of MIP-213(Al) showing the phase purity of the sample.

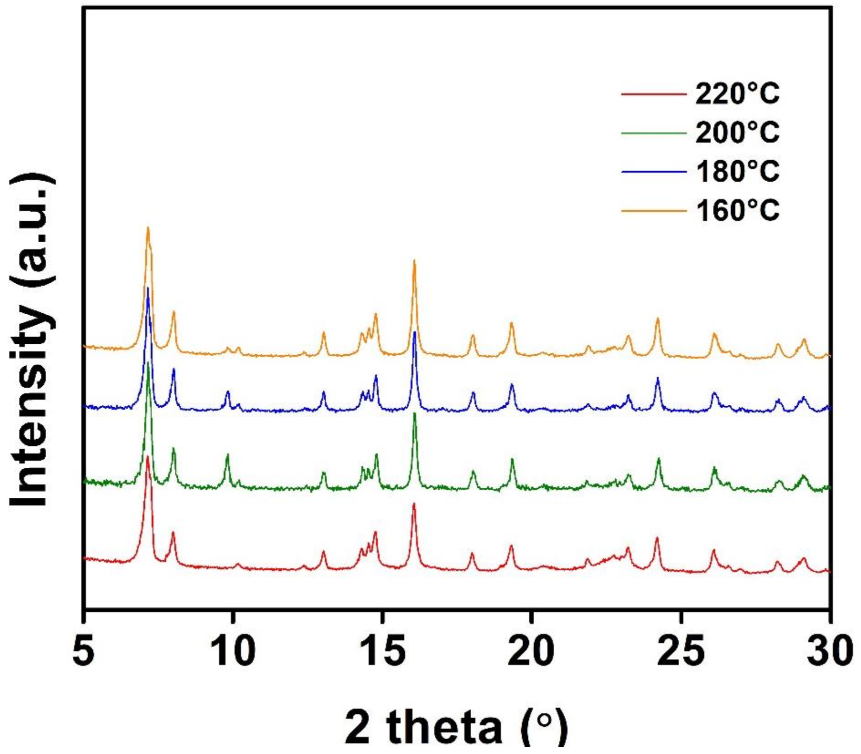

Supplementary Figure 4. A comparative PXRD plot of MIP-213(Al) showing the synthesis temperature optimization process. The MOF could be obtained at temperature range from 160-220°C. Below 160°C the MOF does not form.

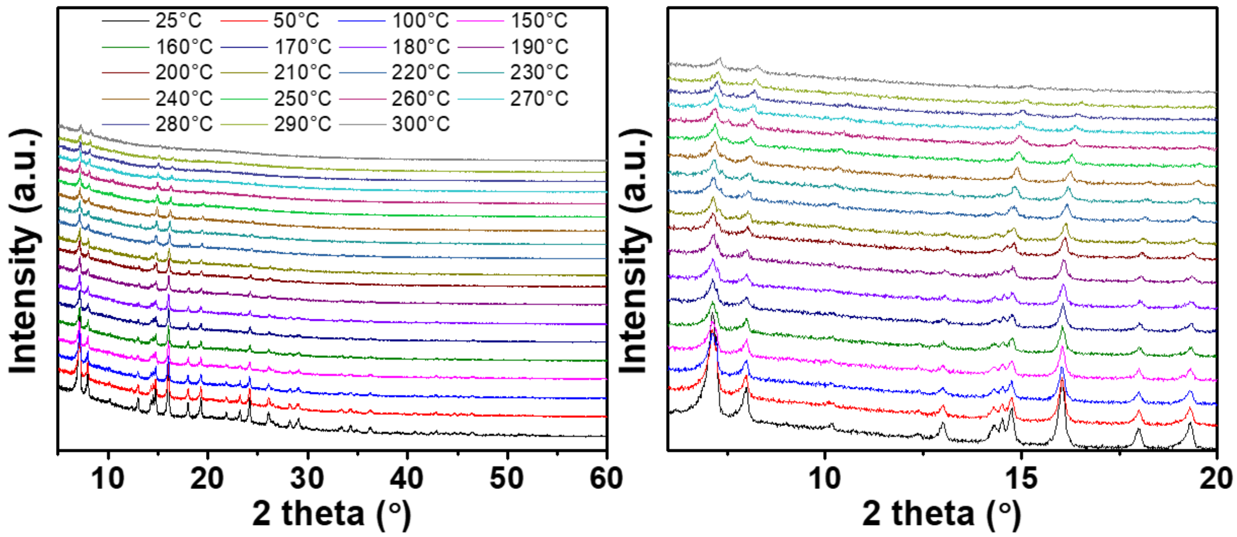

Supplementary Figure 5. (Left) A comparative PXRD plot of the MIP-213(Al) collected at different temperature. Note the sample is thermally stable up-to 250°C. (Right) A zoomed in view of the left picture.

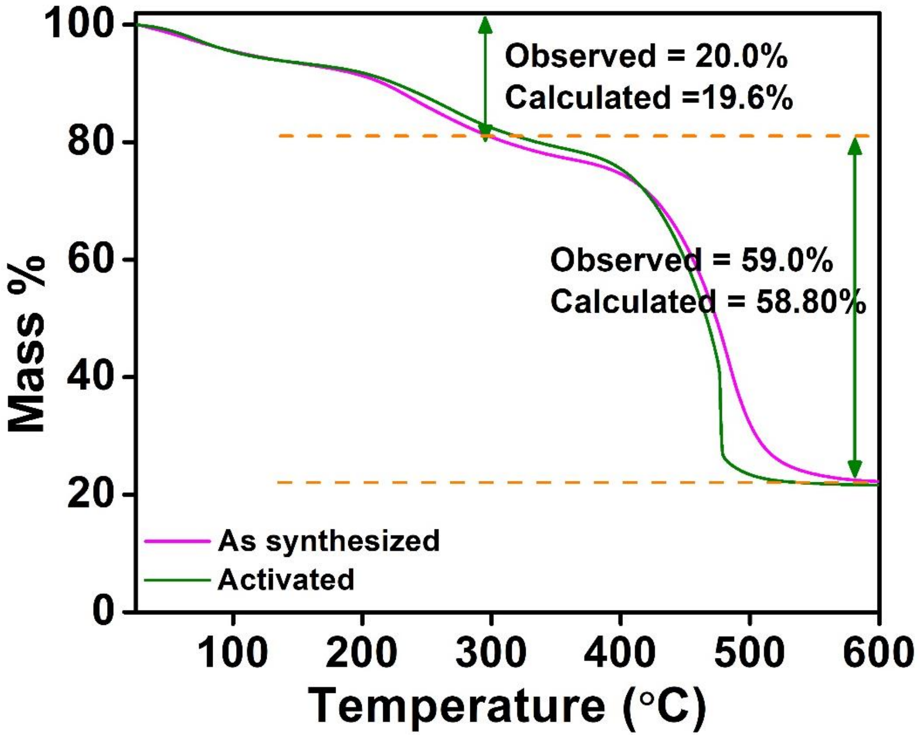

Supplementary Figure 6. TGA curve of MIP-213(Al) showing thermal stability of the framework. Note the sample is thermally stable up to 250°C which was also seen in the variable temperature XRD data. It also confirms that the framework does not contains any free linker in the pore. The TGA data matches well with the chemical formula $[Al_{18}(\mu^2\text{-}OH)_{24}(OH_2)_{12}(MDIP)_6]6Cl.\ 6H_2O$.

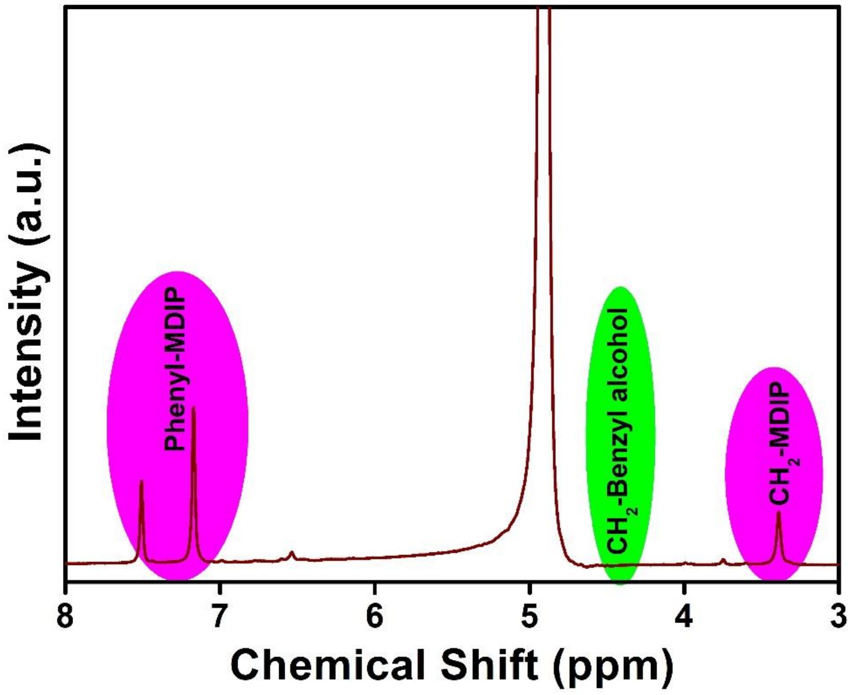

Supplementary Figure 7. ^{1}H NMR spectra of MIP-213(Al) digested in D_2O/NaOH. This clearly show that there are no trapped benzyl alcohol molecules in the MOF.

3. Supplementary Structure solution:

Supplementary Table 1: Selected parameters of two cRED datasets.

**Dataset no.**	**1**	**2**
Rotation range (°)	63.81	40.61
Integration angle (°)	0.142	0.139
Exposure time (s)	0.0125	0.0125
Rotation speed (° s^{-1})	3.7	3.7
Total collection time (s)	18.38	10.96
Resolution (Å)	0.833	0.713
Completeness (%)	97.5	87.2

Supplementary Table 2: Data collection and structure refinement details.

Chemical Formula	C_{51} Al_9 O_{42} ·2.4(Cl) · 12($O_{0.50}$)·O)
Formula weight	1724.41
Crystal system	Hexagonal
Space group	$P6_3/mmc$
a, c (Å)	14.2399(3), 24.1973(7)
V (Å ^{3})	4317(6)
Z	2
Radiation type	Electrons λ = 0.0251 Å
Diffractometer	JEOL COM, F200
No. of measured, independent and observed [I > 3σ(I)] reflections	13125, 1492, 1157
$(\sin \theta/\lambda)_{max}$ (Å $^{-1}$)	0.702
R[F2 > 2σ(F2)], wR(F2), S	0.333, 0.332, 14.70

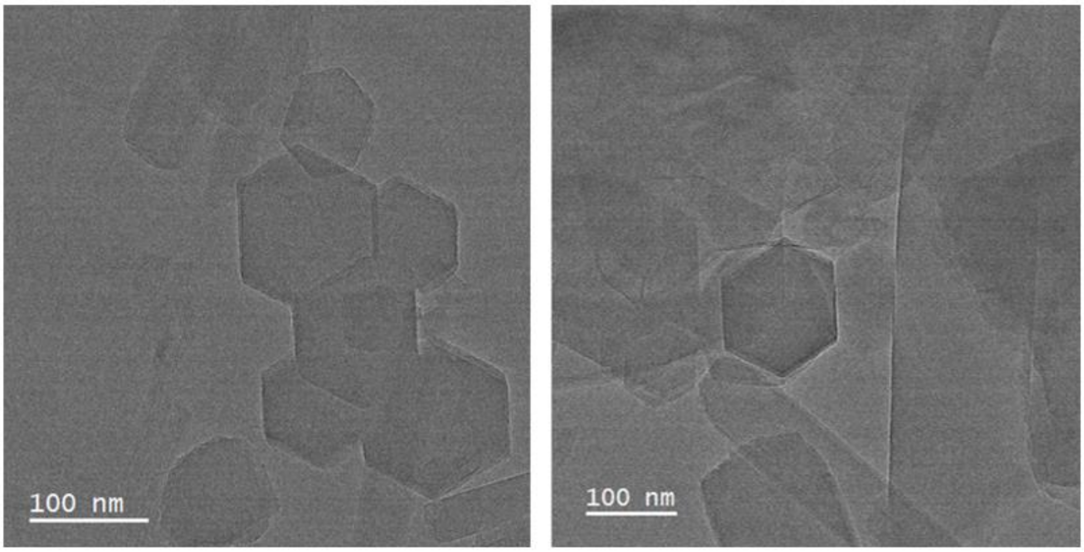

Supplementary Figure 8: TEM images of MIP-213(Al) nano crystals.

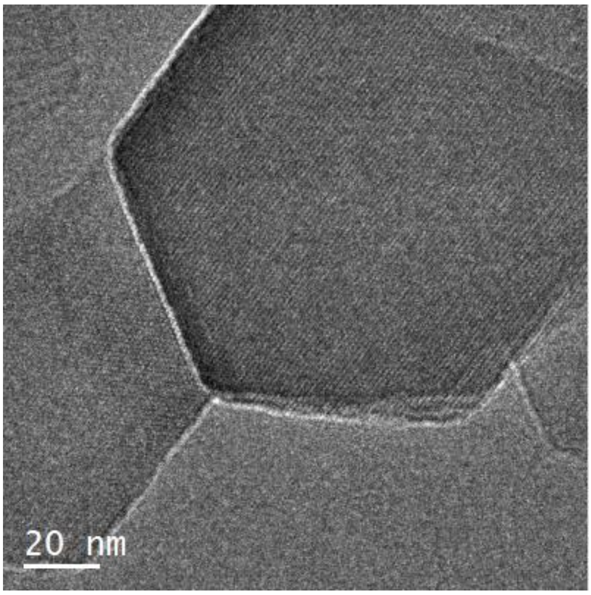

Supplementary Figure 9: Raw HRTEM image of a MIL-213(Al) particle oriented along the [001] axis.

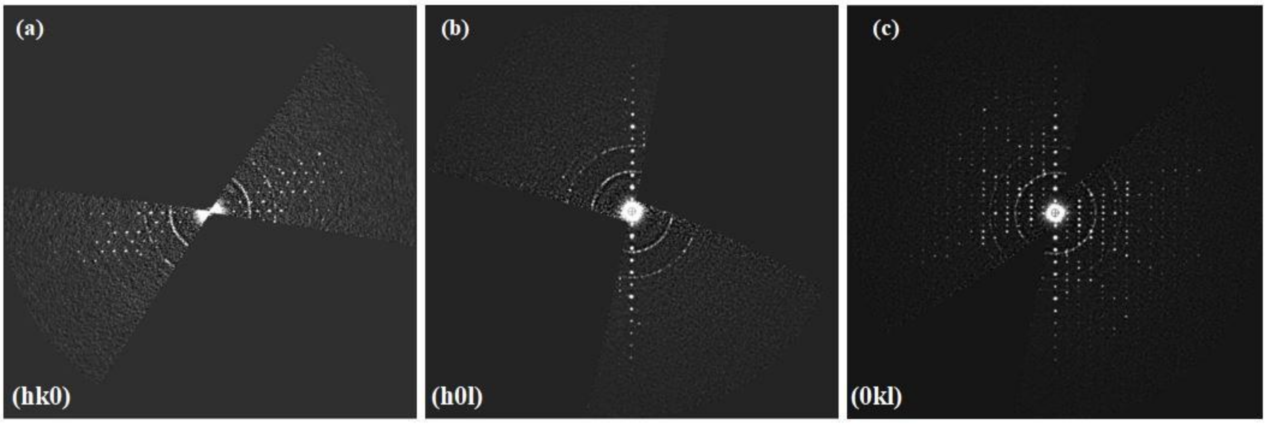

Supplementary Figure 10: 2D slices of the 3D reciprocal space in the (a) *hk*0, (b) *h*0*l* and (c) 0*kl* orientations obtained from the experimental cRED data. Note that the rings observed here are due to ice formation while using the cryo-holder.

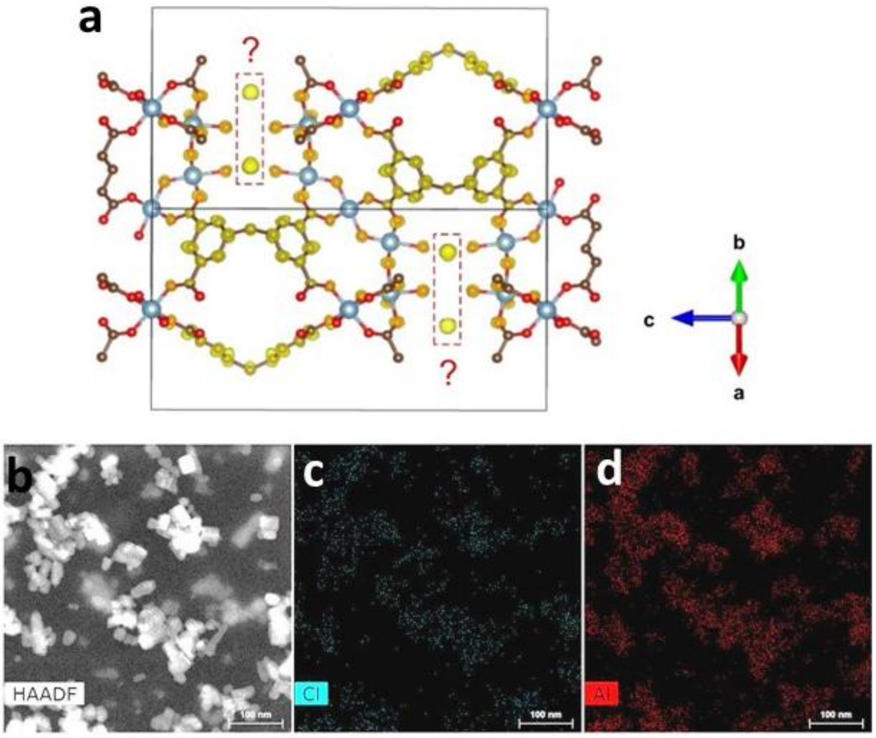

Supplementary Figure 11. (a) Projection of the density (yellow) map generated as an output of the charge-flipping structure solution procedure based on 3D ED data. The deduced atomic model of MIP-213(Al) is overlaid. Color code: Al is shown in light blue, C in brown and O in red. Atoms shown are those assigned first in structure solution. The large unassigned residual densities located in red dashed rectangle was later attributed to Cl^{-} counter anion. (b, c & d) High-angle annular dark-field (HAADF) image of MIP-213(Al) particles and corresponding EDX chemical mapping, respectively, exhibiting the distribution of aluminum and chloride ions.

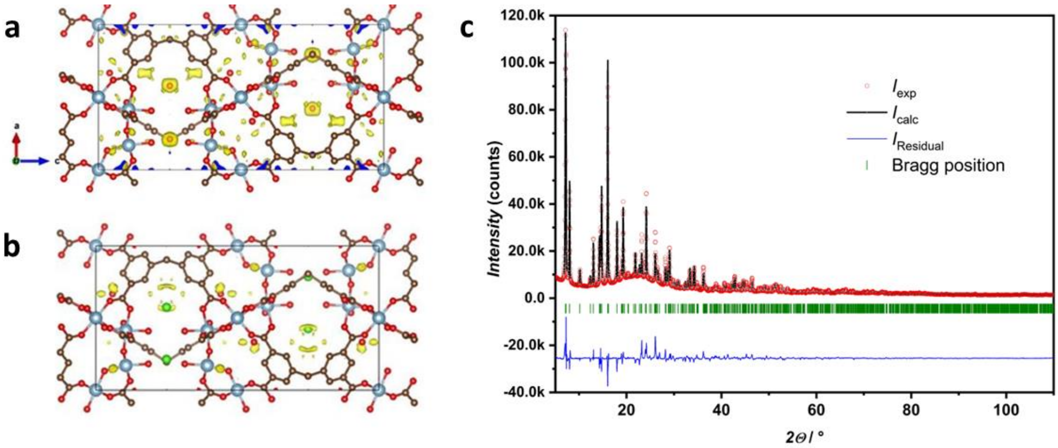

Supplementary Figure 12: Difference Fourier map (yellow) calculated for the MIP-213(Al) structure along the [0 1 0] axis, where the highest residual Q-peak is assigned to (a) the oxygen of a free water molecule and (b) a chloride. Color code: Al is shown in blue, C in brown, O in red and Cl in green. Isosurface level = 0.4 (3σ level) for (sin(θ)/λ)max = 0.5. (c) Rietveld refinement of the structural model

of MIP-213(Al) obtained by 3DED against the high-resolution PXRD pattern (Cu Kα1 radiation λ = 1.5406 Å).

Supplementary Table 3: Unit cell parameters obtained from low dose HRTEM image.

hkl	do (nm^{-1})	SD (%)	d (Å)	a=b= (Å)	a_{model} (Å)
010	0.807	1.036	12.392± 0.128	14.309± 0.148	14.3000

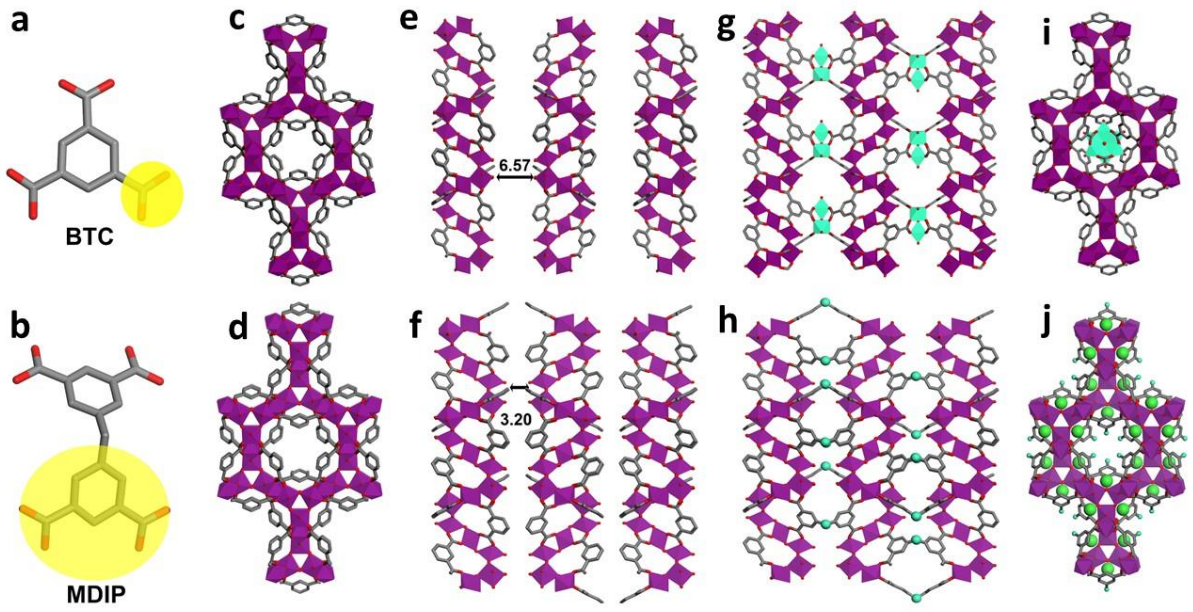

Supplementary Figure 13. A comparison between MIL-96(Al) and MIP-213(Al) crystal structures: (a, b) 3D representation of the BTC and MDIP present in MIL-96(Al) and MIP-213(Al) respectively. (c, d) The similar SBU layers build from the infinite chains of $AlO_4(OH)_2$ and $AlO_2(OH)_3(H_2O)$ /$AlO_2(OH)_4$ octahedra forming a honeycomb lattice based on 18-membered rings present in both the MOFs; (e, f) Cross-sectional view of the SBU layers presented in C and D. (g) 3D view of the crystal structure of MIL-96(Al) along [1 0 0] showing that the SBU layers are connected via the third arm of the BTC linker connecting to isolated μ_3-oxo-centered Al-trimers presented by the cyan polyhedra. (h) 3D view of the crystal structure of MIP-213(Al) along [1 0 0] showing that the SBU layers are connected via the methylene carbon presented by the cyan balls. (i) 3D view of the crystal structure of MIL-96(Al) along [0 0 1]. (j) 3D view of the crystal structure of MIP-213(Al) along [0 0 1]. Al polyhedra, carbon, oxygen atoms are in purple, grey and red, respectively (hydrogen atoms are omitted for clarity).

4. Supplementary Solid-State NMR:

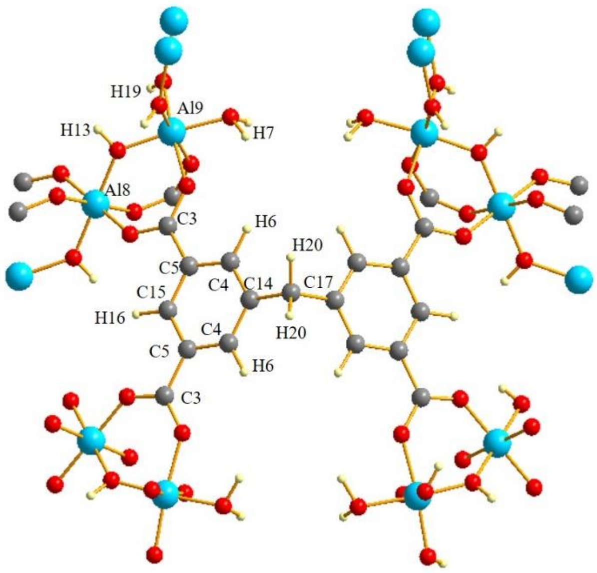

Supplementary Table 4: Isotropic chemical shifts, quadrupolar coupling constant and asymetry obtained by DFT calculations performed on the proposed structure of MIP-213 Al, compared with experimetnaly derived ones and quadrupolar coupling constant and asymetry are given for ^{27}Al. Then Wyckoff intensities (i.e., relative populations of the cristallographic sites) are also given.

Atoms	DFT δ_{iso} (ppm)	Exp. δ_{iso} (ppm)	DFT C_Q (MHz)	C_Q (MHz)	DFT η	η	Wyck.
C3	166.6	169.69					24
C4	133.6	134.10					24
C5	131.0	132.05					24
C14	142.5	140.83					12
C15	131.1	130.55					12
C17	43.8	40.91					6
H6	8.1	9.15					24
H7	8.1	8.36					24
H13	2.1	2.78					12
H16	7.9	6.89					12
H19	1.9	2.08					12
H20	4.1	3.70					12
Al8	3.9	2.43	-8.65	7.8	0.21	0.1	12
Al9	7.9	6.53	1.24	2.4	0.48	0.4	24

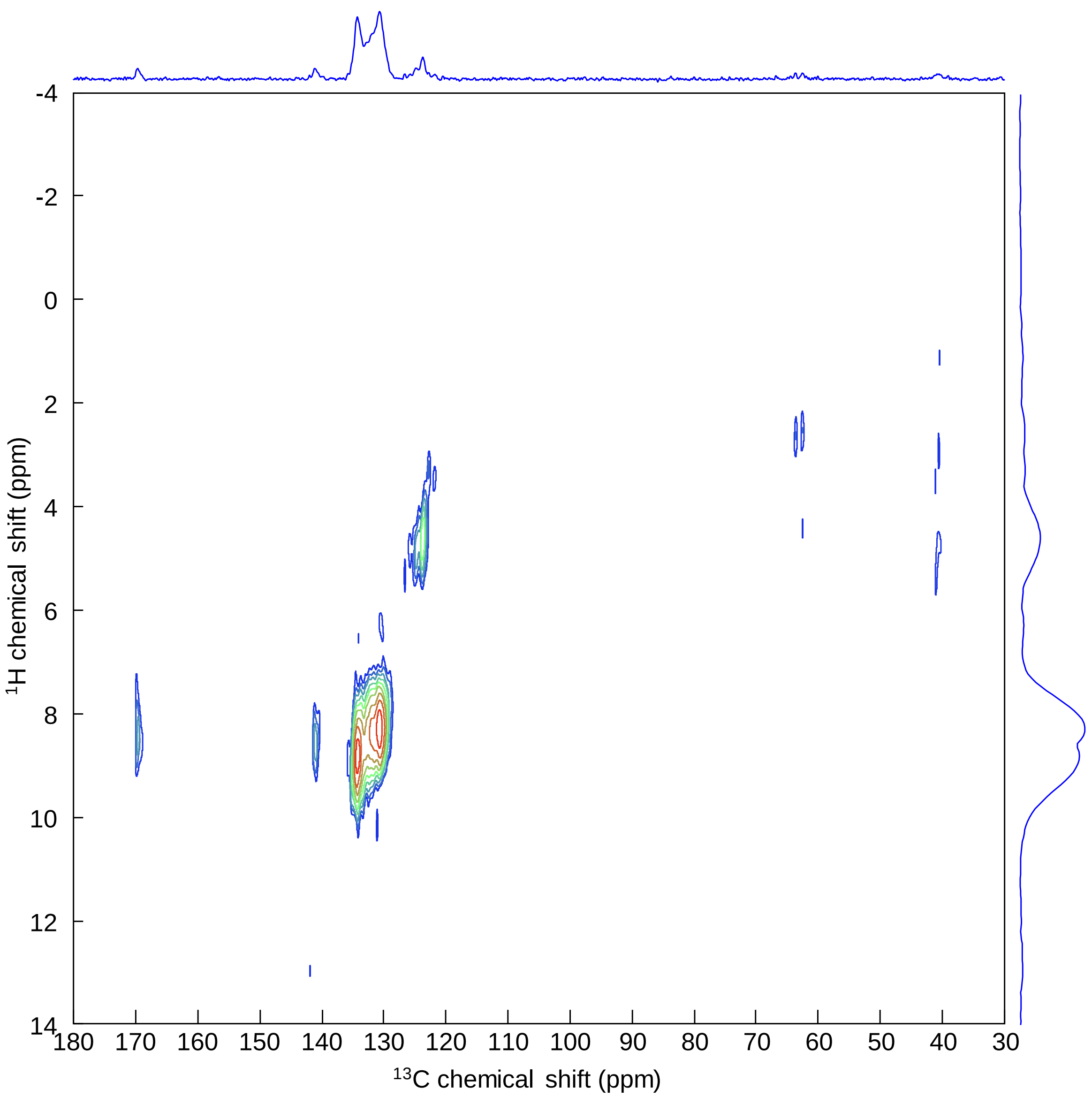

Supplementary Figure 14: ^{1}H-^{13}C NMR HETCOR spectra of MIP-213(Al) dried at 150°C under vacuum for 12h and obtained with a short contact time of 250 µs. 1D side spectra is obtained using Hahn echo for ^{1}H (vertical) and ^{13}C{^{1}H} CPMAS for ^{13}C (horizontal).

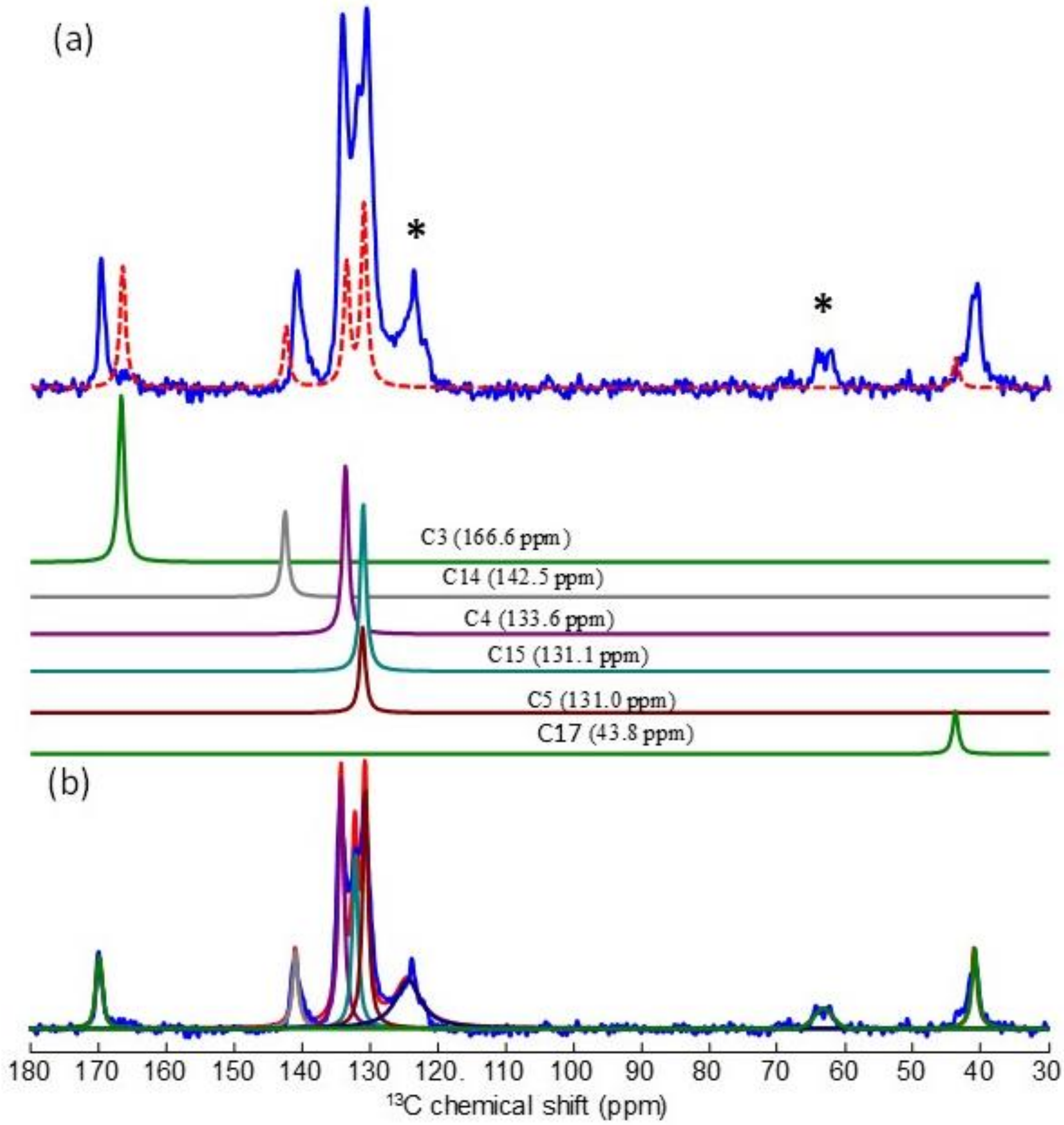

Supplementary Figure 15: (a) $^{13}C\{^{1}H\}$ CPMAS spectra at 250 µs of contact time (blue) along with full DFT-derivated simulation (red) and individual components of DFT-derived simulation and (b) experimental $^{13}C\{^{1}H\}$ CPMAS spectra at 250 µs of contact time (blue) with fit of different inquivalents carbons. Resonances observed around 120 ppm and 60 ppm (indicated by *) belongs to residual solvents (benzyl acohol).

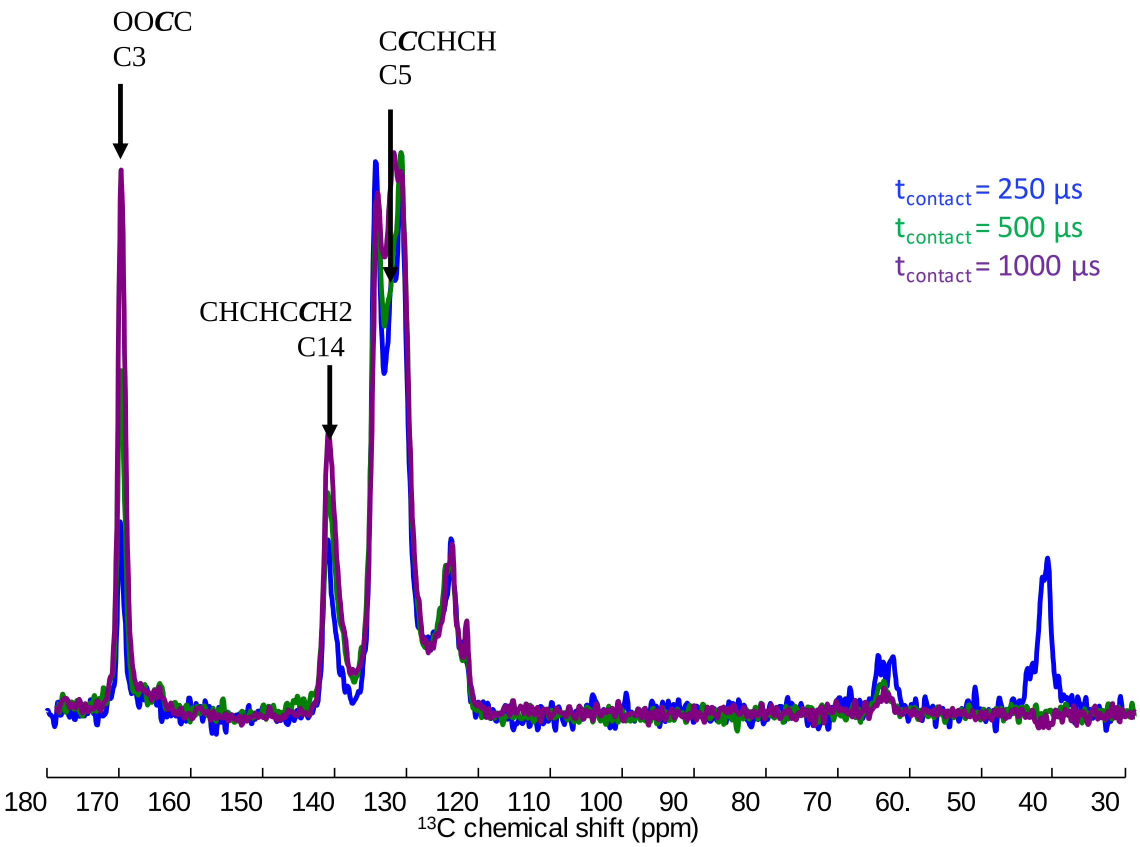

Supplementary Figure 16: $^{13}C\{^{1}H\}$ CPMAS NMR spectra with various contact time, 250 µs (blue), 500 µs (green) and 1000 µs (purple).

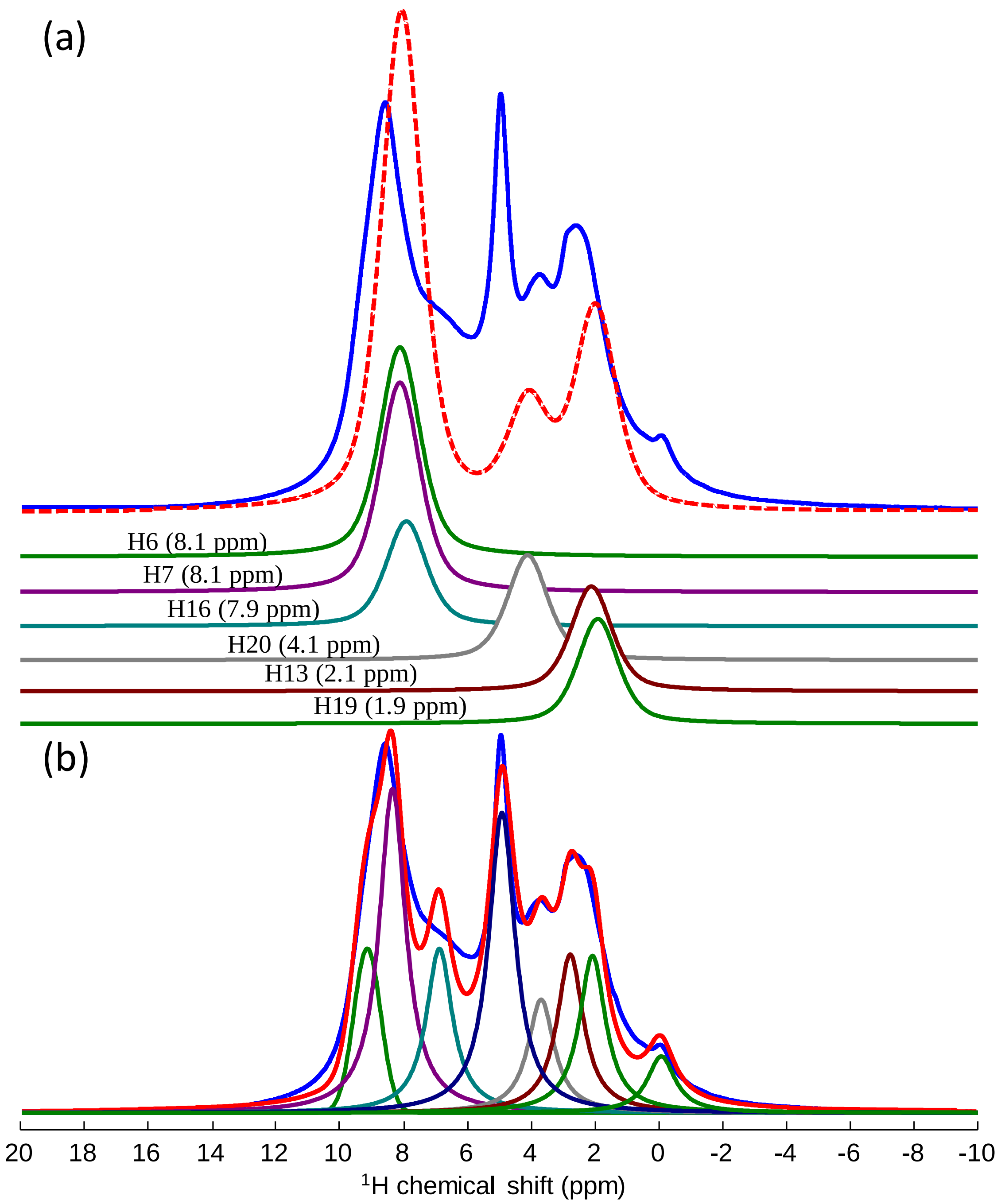

Supplementary Figure 17: (a) Experimental ^{1}H Hahn Echo spectra (blue) along with full DFT-derivated simulation (red) and individual components of DFT-derived simulation and (b) experimental ^{1}H Hahn Echo spectra spectra (blue) with fit of different inquivalents carbons.protons.

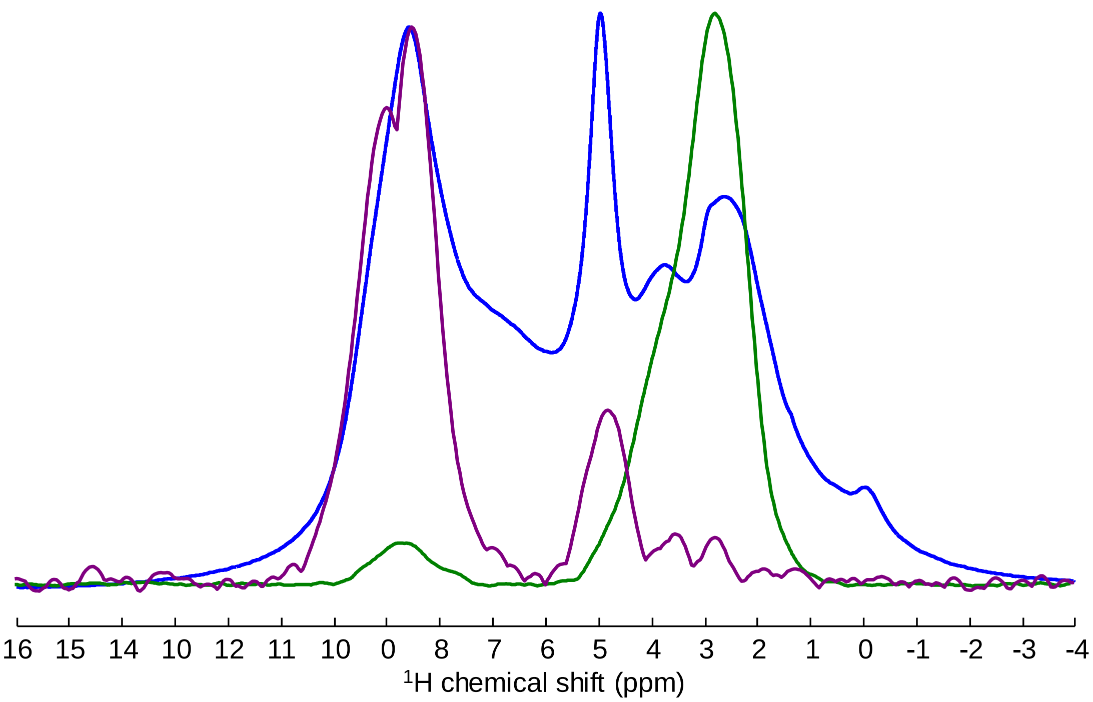

Supplementary Figure 18: ^{1}H Hahn Echo (blue) NMR spectra, positive projection from ^{13}C{^{1}H}CPMAS HETCOR at 250 µs of contact time (purple) and positive projection from ^{27}Al{^{1}H} D-HMQC at 400 µs of recoupling (green).

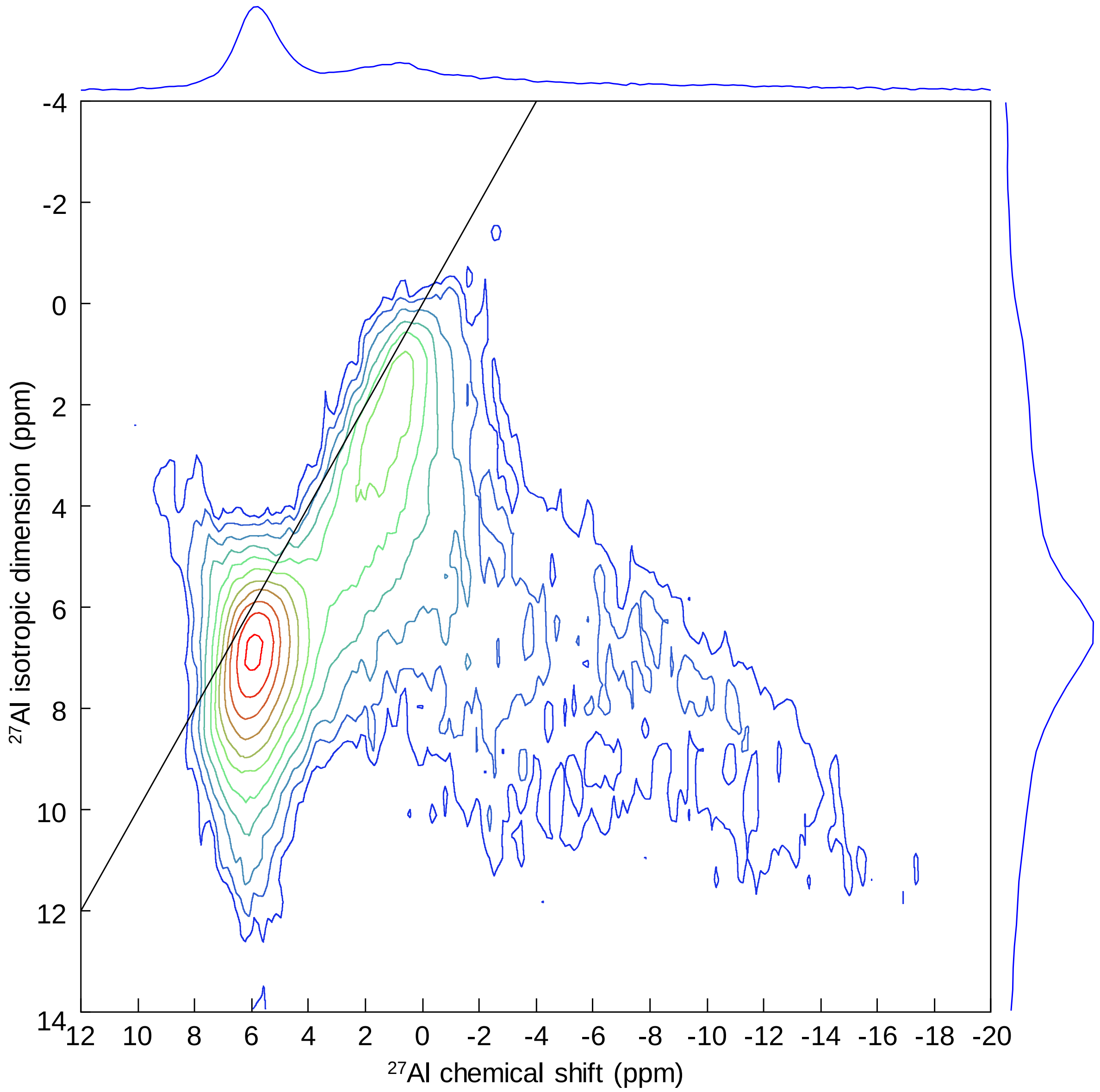

Supplementary Figure 19: ^{27}Al-^{27}Al NMR MQMAS spectra of MIP-213(Al) dried at 150°C under vacuum for 12h.

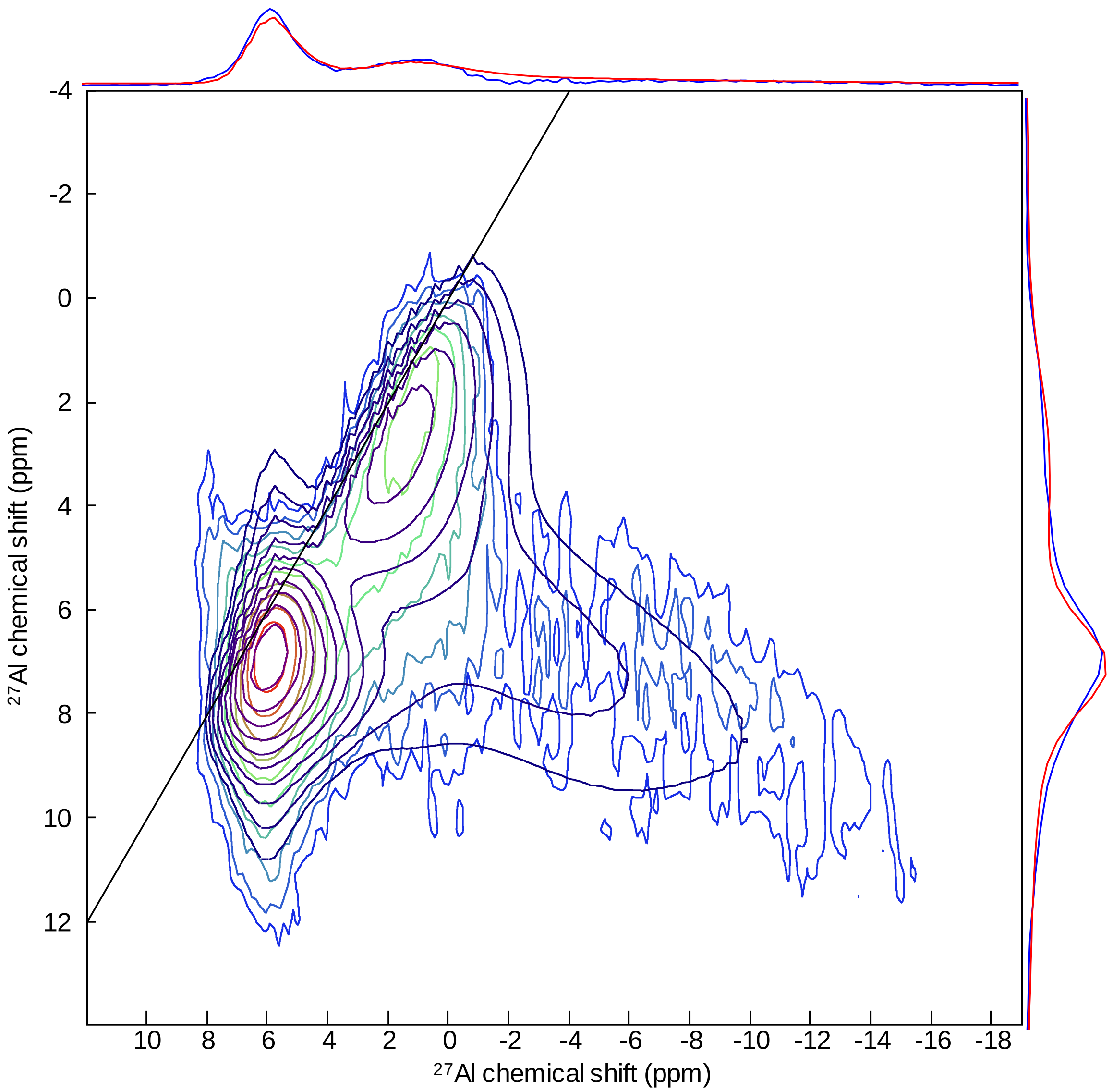

Supplementary Figure 20: ^{27}Al-^{27}Al MQMAS spectrum and fit with 3 sites of MIP-213 Al.

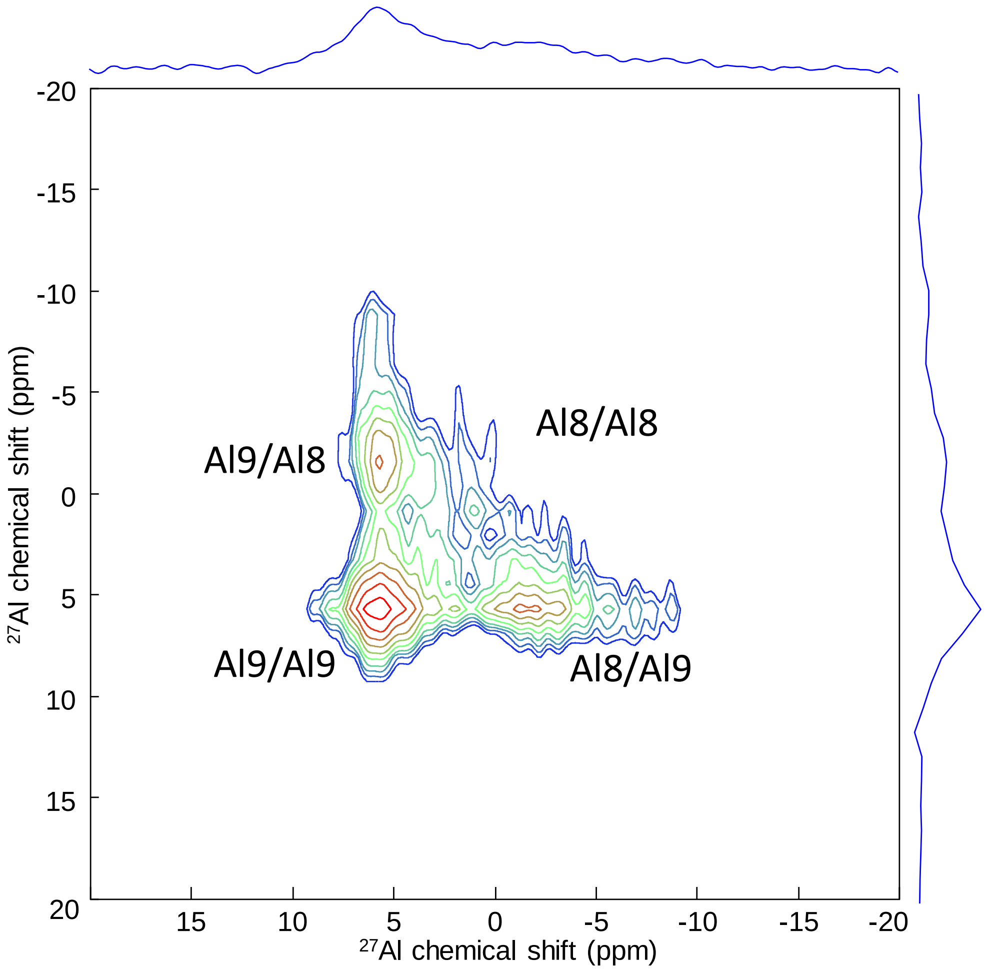

Supplementary Figure 21: ^{27}Al-^{27}Al sheared and symmetrized 2Q/1Q experiment.

5. Supplementary First-Principle Calculations:

First principles calculations with periodic boundary conditions were performed using the CASTEP code[5,6] which employs the planewave pseudopotential formalism of Kohn–Sham DFT. The electron correlation effects are modeled using the semi-local Perdew−Burke−Ernzerhof (PBE) exchange–correlation functional using D2 dispersion corrections by Grimme et al.[7] The generalized gradient approximation (GGA)[8] was used along with the default "ultrasoft"[9] pseudopotentials of CASTEP 20.11 and a planewave cut-off energy of 500 eV. Starting from the experimentally determined crystal structure, only atomic positions were fully relaxed with convergence thresholds set to 5.0×10^{-7} eV/atom for the total energy. The NMR calculations were performed using the Gauge Including Projector Augmented Wave approach (GIPAW)[10,11] at the same cut-off energy of 500 eV.

Calibration of the ^{13}C and ^{1}H chemical shifts were obtained by performing the calculations on amino-acids according to the above-described conditions and compare those to known experimental values. This led to $\delta(^{13}C) = -0.9589\ \sigma(^{13}C) + 165.541$ ($R^2 = 0.999$), and $\delta(^{1}H) = -0.8483\ \sigma(^{1}H) + 26.534$ (R^2 =

0.978). ^{27}Al calibration has been obtained using corundum and gibbsite, leading to $\delta(^{27}Al) = -0.820\ \sigma(^{27}Al) + 454.601$ ($R^2 = 0.893$).

6. Supplementary Gas Sorption Analysis:

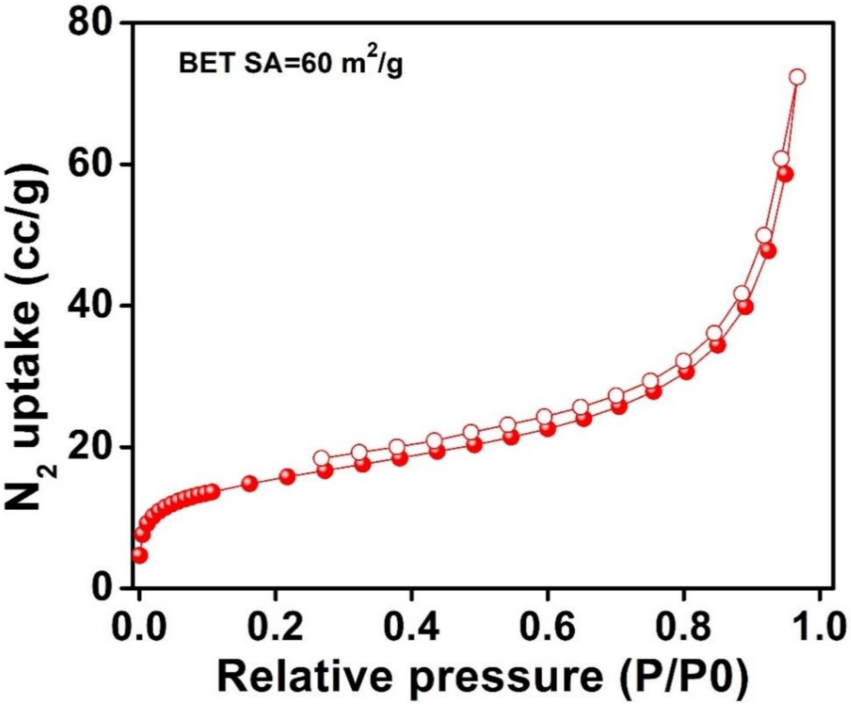

Supplementary Figure 22. N_2 isotherm of MIP-213(Al) collected at 77K. Note: The material does not adsorb much N_2 as expected from the narrow pores present in the material.

Langmuir Fits:

All the isotherms were fitted to the Single-Site Langmuir (SSL) equation as presented below. During the investigation, we also used modified Langmuir equations to take care of substantial errors in the model. It is well established that even a small error in the fitting can lead to over estimation or under estimation of the selectivity values.

All the pure component isotherms were fitted via solving the Langmuir equation using the solver function embedded in Microsoft Excel. We used the same procedure established by Keller *et al.*[12] This offers a balanced approach against the issues associated with favouring either high or low pressure regions of the Langmuir equation[13].

Single-Site Langmuir (SSL):

$$q_i = q_m \frac{K_i P}{1 + K_i P}$$

Dual-Site Langmuir (DSL):

$$q_i = q_{m,1} \frac{K_1 P}{1 + K_1 P} + q_{m,2} \frac{K_2 P}{1 + K_2 P}$$

Ideal Adsorbed Solution Theory (*IAST*):

We performed the IAST calculations as defined by Prausnitz *et al.*[14] The selectivity equation that was followed is given below.

Selectivity:

$$S_{1,2} = \frac{q_1/q_2}{P_1/P_2}$$

IAST Fitting parameters for MIP-213(Al):

GAS A: CO_2

GAS B: N_2

Gas Mixture		Intrinic Selectivity	
YA =	0.15	α_1 =	243.331299
YB =	0.85		

Gas A Constants		Gas B Constants	
qA1 =	2.405896484	qA1 =	1.99494779
qA2 =	0	qA2 =	0
kA1 =	0.005029723	kA1 =	2.4928E-05
kA2 =	0	kA2 =	0
na1 =	0.837407901	na1 =	1.16781412
na2 =	0	na2 =	0
HA1 =	0.012100994	HB1 =	4.9731E-05
HA2 =	0	HB2 =	0

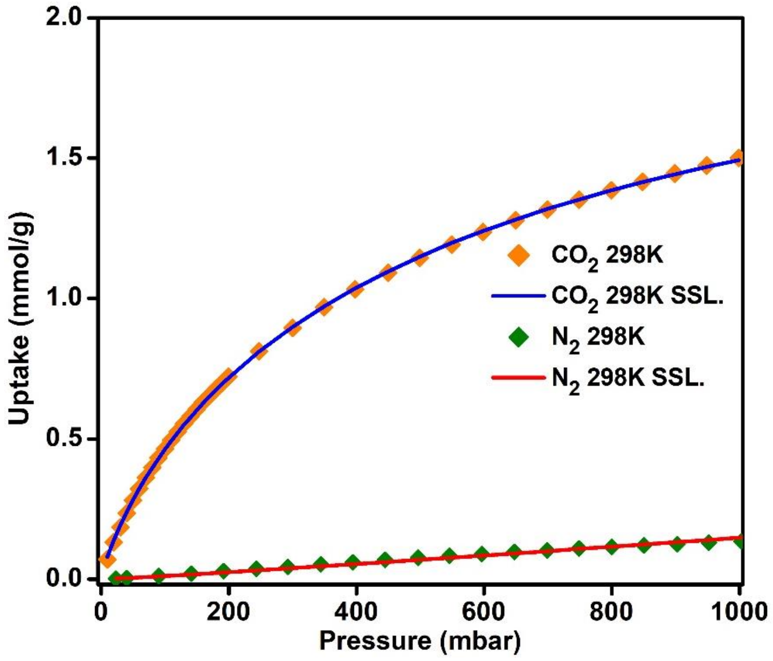

Supplementary Figure 23. *IAST* fitting of CO_2 and N_2 isotherms for MIP-213(Al) collected at 298K.

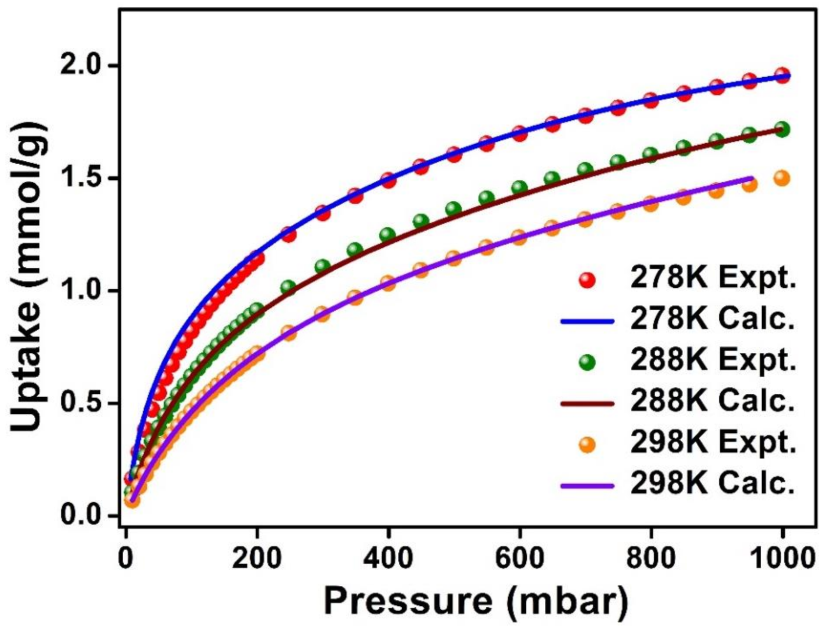

Supplementary Figure 24. Comparison of experimental isotherms of MIP-213(Al) to the ones obtained from virial modelling carried out using CO_2 isotherms collected at 278, 288 and 298K.

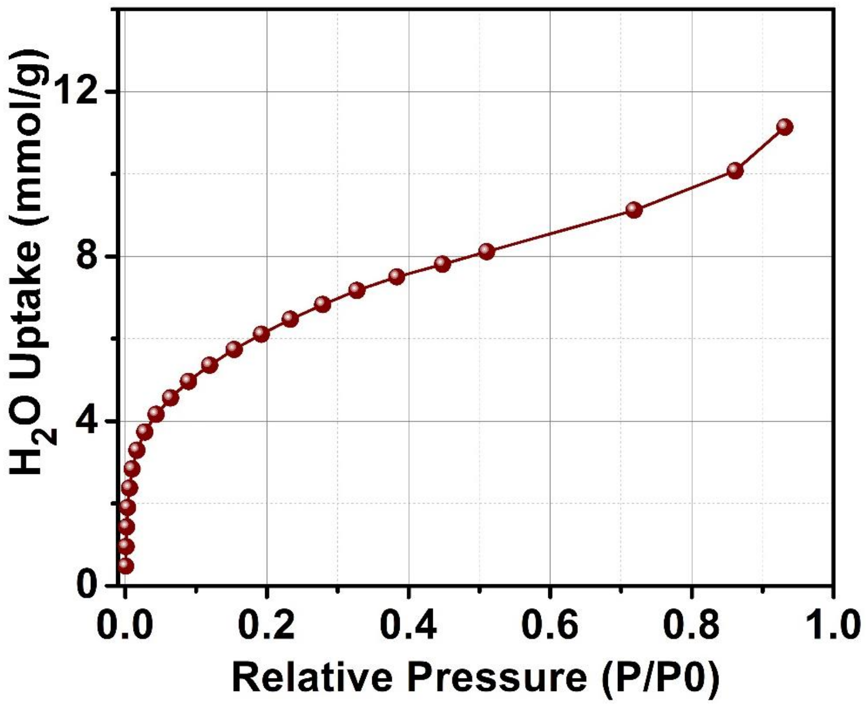

Supplementary Figure 25. Water isotherm of MIP-213(Al) collected at 298K shows that the material is neither too hydrophilic nor too hydrophobic.

7. Supplementary Hydrolytic Stability:

To test the hydrolytic stability of the MOF we have subjected the sample under boiling water (2 mg of sample per 1 ml water) condition for 24 hours. After that the sample was recovered via filtration and dried. The water boiled sample was then characterized using the PXRD and sorption analysis.

8. Supplementary Note on Ion Exchange of The Material:

As the framework is cationic and contains some chloride ions are required to balance the overall charge of the framework, it indeed calls for ion exchange trials. Therefore, we were keen to check this possibility and performed many experiments to exchange the chloride ions with other halides. However, the chloride ions were not exchanged with other halides such as bromide, iodide, etc. as confirmed from the characterization of the solid after the exchange experiments (EDX does not show any trace of the exchanged anion). This is probably due the fact that there exists very strong H-bonding interaction between the chloride and the terminal water molecules connected to the Al-trimers. Also, the space available between the Al-trimers

from the adjacent SBU layers offers probably a perfect fit only for the chloride ions. Other than halides we tried unsuccessfully to exchange the chlorides with some oxo anions such as MnO_4^-. But in this case also the chloride ions were not exchanged. Alternative to the exchange process, we tried without any success to produce the framework with other halide by introducing some bromide and iodide in the synthesis mixture. However, we were not successful to get any positive result**.**

Supplementary References:

1. Altomare, A. *et al. J. Appl. Cryst*. **46**, 1231-1235 (2013).
2. Loiseau, T. *et al*. *J. Am. Chem. Soc*. **128**, 10223-10230 (2006).
3. Rodriguez-Carvajal, J. *Physica B*, **192**, 55-69 (1993).
4. Massiot, D. *et al*. *Magn. Reson. Chem*. **40**, 70–76 (2002).
5. Segall, M. D. *et al*. *J. Phys.: Condens. Matter* **14**, 2717 (2002).
6. Clark, S. J. *et al*. *Kristallogr*. **220**, 567 (2005).
7. Grimme, S. J. *Comput. Chem*. **27**, 1787–1799 (2006).
8. Perdew, J. P. Burke, K. & Ernzerhof, M. *Phys. Rev. Lett*. 77, 3865 (1996).
9. Vanderbilt, D. *Phys. Rev. B* **41**, 7892 (1990).
10. Pickard, C. J. & Mauri, F. *Phys. Rev. B* **63**, (2001).
11. Yates, J. R. Pickard, C. J. & Mauri, F. *Phys. Rev. B*, 76 (2007).
12. Kemmer, G. & Keller, S. *Nat. Protoc.* **5**, 267-281 (2010).
13. Richter, E., Schuetz, W. & Myers, A. L. *Chem. Eng. Sci.* **44**, 1609-1616 (1989).
14. Prausnitz, J. M. *et al. AIChE J.* **11**, 121 (1965).
