# Supplementary material for: A robust ultra-microporous cationic aluminum-based metal-organic framework with a flexible tetra-carboxylate linker
Source: Commun Chem. 2023 Jul 6;6:144. doi: 10.1038/s42004-023-00938-x (PMC10326013; doi:10.1038/s42004-023-00938-x)
Supplement: Supplementary file 2 — Description of Additional Supplementary Files [file 42004_2023_938_MOESM2_ESM.docx]

Description of Additional Supplementary Files

**File name:** Supplementary Data 1

**Description: CIF of MIP-213(Al)**

**File name:** Supplementary Data 2

**Description: IAST calculation and related numerical source data**
